# Interleukin-4 from curcumin-activated OECs emerges as a central modulator for increasing M2 polarization of microglia/macrophage in OEC anti-inflammatory activity for functional repair of spinal cord injury

**DOI:** 10.1186/s12964-024-01539-4

**Published:** 2024-03-06

**Authors:** Jianbin Guo, Xiangwen Tang, Peng Deng, Hao Hui, Bo Chen, Jing An, Gaorong Zhang, Kuohao Shi, Jinchao Wang, Yuqing He, Dingjun Hao, Hao Yang

**Affiliations:** 1 Department of Joint Surgery, Hong Hui Hospital, Xi’an Jiaotong University, Xi’an, 710054 China; 2 Basic Medical School Academy, Basic Medical School Academy, Shaanxi University of Chinese Medicine, Xianyang, 712046 China; 3 Translational Medicine Center, Hong Hui Hospital, Xi’an Jiaotong University, Xi’an, 710054 China; 4 Department of Spine Surgery, Hong Hui Hospital, Xi’an Jiaotong University, Xi’an, 710054 China; 5 School of Basic Medical Sciences, Ningxia Medical University, Yinchuan, 750004 Ningxia China

**Keywords:** Activated olfactory ensheathing cells, Interleukin-4, Microglia/macrophage polarization, Neuroinflammation, JAK1/STAT1/STAT3/STAT6

## Abstract

Microglia/macrophages are major contributors to neuroinflammation in the central nervous system (CNS) injury and exhibit either pro- or anti-inflammatory phenotypes in response to specific microenvironmental signals. Our latest in vivo and in vitro studies demonstrated that curcumin-treated olfactory ensheathing cells (aOECs) can effectively enhance neural survival and axonal outgrowth, and transplantation of aOECs improves the neurological outcome after spinal cord injury (SCI). The therapeutic effect is largely attributed to aOEC anti-inflammatory activity through the modulation of microglial polarization from the M1 to M2 phenotype. However, very little is known about what viable molecules from aOECs are actively responsible for the switch of M1 to M2 microglial phenotypes and the underlying mechanisms of microglial polarization. Herein, we show that Interleukin-4 (IL-4) plays a leading role in triggering the M1 to M2 microglial phenotype, appreciably decreasing the levels of M1 markers IL‑1β, IL‑6, tumour necrosis factor-alpha (TNF-α) and inducible nitric oxide synthase (iNOS) and elevating the levels of M2 markers Arg-1, TGF-β, IL-10, and CD206. Strikingly, blockade of IL-4 signaling by siRNA and a neutralizing antibody in aOEC medium reverses the transition of M1 to M2, and the activated microglia stimulated with the aOEC medium lacking IL-4 significantly decreases neuronal survival and neurite outgrowth. In addition, transplantation of aOECs improved the neurological function deficits after SCI in rats. More importantly, the crosstalk between JAK1/STAT1/3/6-targeted downstream signals and NF-κB/SOCS1/3 signaling predominantly orchestrates IL-4-modulated microglial polarization event. These results provide new insights into the molecular mechanisms of aOECs driving the M1-to-M2 shift of microglia and shed light on new therapies for SCI through the modulation of microglial polarization.

**Supplementary Information:**

The online version contains supplementary material available at 10.1186/s12964-024-01539-4.

## Introduction

Accumulating evidence suggests that microglia-initiated neuroinflammation is envisioned as a significant and inevitable pathological process that occurs during various types of damages to the central nervous system (CNS) and the different neurodegenerative diseases [1–5]. In general, a wide spectrum of consecutive pathological events occurs following acute neurological insults, leading to a progressive exacerbation of the initial injury [6]. Among all the mechanisms responding to subsequent damage, the inflammatory response functions as one of the critical processes that increase SCI severity and the occurrence of numerous sequelae after neural injury [6–9]. In particular, neuroinflammatory stress usually exacerbates the microenvironment deterioration in the lesion area, resulting in more severe cell and tissue damage [10–12]. Therefore, to achieve an efficient therapy strategy for the treatment of neuronal regeneration, it is essential to promptly reverse the growth-inhibiting microenvironment created by neuroinflammation. The microglia/macrophages involved in neuroinflammation following SCI are the main inflammatory cells in the CNS [10–12]. Normally, microglia are principle innate immune cells and exist in a relatively quiescent surveillance state in the CNS [13–15]. Once a pathological or physiological insult occurs in the CNS, microglia can become activated and dynamically and temporally develop into M1 and M2 phenotypes according to their upstream activating stimuli and downstream effector functions [1, 6, 11, 15, 16]. The M1 phenotype microglia release proinflammatory mediators (IFN-γ, IL-1β, TNF-α, IL-6, CXCL10, etc.), produce reactive oxygen species (ROS) and NO, and activate proteolytic enzymes (MMP9, MMP3), all of which elicits detrimental effects on neurons and further exacerbates the extent of the initial neural injury and associated dysfunction [11, 17–22]. In contrast, M2 microglia release anti-inflammatory cytokines (IL-10, TGF-β, IL-4, IL-13 and IL-1Ra) and bioactive factors (IGF-1, VEGF, PDGF, GDNF and BDNF, etc.), which suppresses the proinflammatory responses and facilitates neural recovery and remodeling [6, 11, 21, 22]. It can be inferred that the phenotype of microglia in the injured CNS is closely related to postinjury neuroinflammation, and secondary damage severity and functional recovery. Therefore, inhibition of M1 microglial polarization along with enhancement of M2 microglial polarization is likely to be a potential therapeutic strategy for the treatment of SCI due to inflammation-related progressive exacerbation of the initial injury.

Olfactory ensheathing cells (OECs), a specialized type of glial cell, have increasingly become promising cell-based therapy candidates for CNS injury. Strikingly, there is compelling evidence that the mechanisms underlying the proregenerative potential of OECs are associated with the immune properties and continuous secretion system signaling responsible for anti-neuroinflammation, immunomodulation, ROS scavenging, and maintenance of the internal microenvironment homeostasis [12, 23–27]. Accordingly, transplanted OECs could mainly orchestrate the crosstalk of several mechanisms to improve neuronal survival and neurite-extension and subsequent functional recovery [12, 23, 28–30]. Although there is still no direct evidence regarding the impact of OEC transplantation on neuroinflammation subsequent to CNS injury, several studies using SCI animal models have demonstrated the association between OECs and inflammation [2, 6, 28, 31–33]. By using several advanced techniques, an in-depth understanding of OEC anti-inflammatory potential is now being promulgated. That is say, OECs can effectively reverse neuroinflammation, mainly due to their unique secretion of anti-inflammatory factors and consequent immunomodulatory effects on immune cells. First, anti-inflammatory cytokines secreted from OECs are capable of modulating the inflammatory response, resulting in a decrease in the production of several proinflammatory factors, such as IL-1β, TNF-α and IL-6, by microglia/macrophages [28, 30, 31, 33]. Moreover, these cytokines also reduce infiltration of immunocytes, thereby effectively attenuating further inflammatory damage. Our latest several studies revealed that several factors released from aOECs effectively promote neural regeneration after SCI by modulating cell survival, proliferation and migration [28, 29, 31]. This is mainly attributed to curcumin-induced enhancement of all OEC positive effects, including phagocytic capacity, anti-neuroinflammation, and promotive effects on neuronal survival and growth apart from their own proliferation and migration. Curcumin, a bioactive polyphenol extracted from rhizome of the *Curcuma longa,* possesses a variety of pharmacological and biological effects properties, such as anti-inflammatory, antioxidant, anticancer, immunomodulatory, autophagy-enhancing, and anti-microbial, etc. [34–38]. In addition to the reported benefits, numerous studies have shown that Curcumin exerts distinct neuroprotective and neutrophic effects on neuronal cells and glia by modulating their related signalling pathways [39–41]. Noteworthy, several recent reports have demonstrated that Curcumin can improve OEC proliferation, migration, morphologic changes, secretion of neurotrophic factors and phagocytic activity [29, 42]. That is, Curcumin significantly enhance the activation of OECs. In this regard, Curcumin potentiates the beneficial behavior of OECs including anti-inflammation and immunomodulation. As a result, aOECs could function as the most promising candidates for cell-based transplantation therapy targeting the CNS injury and neurodegenerative diseases. More importantly, we also found that exosomes released from aOECs could reverse the neuroinflammation following SCI via polarization of M1 to M2 microglia, leading to neural survival and axonal regeneration [24]. Nevertheless, the signaling molecules released from aOECs that mediate the M1 to M2 phenotypic conversion during complicated anti-inflammatory events are still unknown. Moreover, the exact molecular mechanisms underlying the M1 to M2 shift of microglia remain unclear.

In the current study, we sought to examine the effects of OECs activated by curcumin (CCM) on the alterations in microglial M1/M2 polarization, together with molecular and cellular evidence demonstrating that the M1/M2 phenotype switch in microglia is mainly triggered by IL-4 released from aOECs, thus leading to neuronal outgrowth. Notably, this effect was abated by IL-4-siRNA or administration of anti-IL-4 neutralizing antibody (NTAb). In addition, we demonstrated that IL-4 activates JAK1/STAT1/3/6-targeted downstream signals via the IL-4 receptor and cross-talks with NF-κB/SOCS1/3 signaling, all of which are likely to contribute to M1/M2 polarization. Of unusual significance, the role of IL-4 released from aOECs in regulating microglial polarization and the molecular mechanisms involved in M1 to M2 phenotypic conversion may provide insight into how the innate immune response can be modulated during distinct phases of injury to promote neuroprotection and neurorepair.

## Materials and methods

### Primary culture of OECs, microglial cells and neurons

All experimental procedures conducted on animals were approved by the Animal Experimentation Ethics Committee of Xi’an Jiaotong University and were in accordance with the China Code of Practice for the Care and Use of Animals for Scientific Purposes. Primary OECs were prepared from Sprague–Dawley (SD) rat olfactory bulbs at 2.5 months of age and further purified according to a previously reported methods by Yang with slight modifications [26]. Briefly, the outer nerve fiber and granular layers of olfactory bulbs were dissected from rats and digested in a 0.25% trypsin and 0.2% dispase II/phosphate-buffered solution for 25 min at 37 °C. The cell suspension was obtained by repetitive trituration of the digested tissue with a 1 ml fire-polished Pasteur pipette, and plated on PLL-coated culture flasks and cultured in DMEM/F12 with 10% FBS at 37 °C in a humidified incubator at 5% CO_2_. When the OECs reached 85%–90% confluence, cell purification was conducted by differential cell adhesiveness and maintained in DMEM/F12 media containing 1% G5 supplement for further experiments.

As for microglia, primary microglial cultures were prepared from cortex of embryonic 15- to 17-day-old SD rats as described previously with minor modifications [31]. Cortical tissues were dissected, minced, and dissociated by mechanical trituration using a fire-polished Pasteur pipette, followed by enzymatization with 0.125% trypsin and 50 U/ml DNAse I. Concomitantly, cortical cells were suspended, mixed, plated in poly-lysine (PLL)-coated 25 cm^2^ culture flasks at a density of 2 × 10^5^ cells/cm^2^ in 4 ml DMEM with 10% FBS, 100 U/ml penicillin and 100 μg/ml streptomycin, and maintained at 37 °C in a humidified atmosphere of 5% CO_2_. At 7 to 10 days, culture flasks were then placed on an orbital shaker at 190 rpm for 5 h. The media containing detached cells was harvested and centrifugated at 1000 rpm for 10 min. Thereafter, a cell pellet was acquired and re-suspended in the same media as described above. After 4–8 h of culture, highly pure microglial cells were obtained by shaking cross in hand at room temperature (RT) for 5 min, and replated on culture flasks and glass coverslips to identify the cell purity. When the purity of microglial cells reached over 85%, cells were used for further experiments.

As for spinal cord neuron cultures, primary neurons were prepared from the spinal cords of embryonic 12- to 14-day-old SD rats as previously described by Yang et al. [29, 43]. Briefly, the spinal cords were dissected and were trypsinized with 0.125% (w/v) trypsin at 37 °C for 25 min prior to mechanical trituration. The dissociated cells were then collected by centrifugation and diluted to an appropriate cell density of 1 × 10^5^ cells/cm^2^ with Neurobasal medium supplemented with 2% B27, and plated into either 6-well culture plates or coverslips coated with PLL. Cultures were maintained at 37 °C in a humidified atmosphere of 5% CO_2_. The cultures were maintained and prepared for next experiments.

### Activation of OECs and collection of conditioned culture medium

To examine the impact of aOECs on the M1/M2 phenotype switch in microglia and the roles of viable molecules secreted from aOECs in this process, OECs were reseeded on coverslips, 35-mm dishes, and 96-well plates, and subsequently treated as previously described by Yang et al. [27]. Noteworthy, 1 μM curcumin was used to activate OECs in the present study. Activated OECs-conditioned medium (aOECCM) was collected and processed according to our previously described method [29, 44]. The cultures were maintained and prepared for the following experiments.

### Assessment of activated OECs

The activation of OECs was evaluated according to our previously described methods. Briefly, the mRNA expression levels of Cxcl1, TLR4, TG2, and PSR were determined by quantitative real-time PCR (qRT‒PCR) and western blot assay according to the following description, respectively. In addition, the proliferative capacity of OECs was evaluated by BrdU incorporation array and MTT assay. The identification of OEC activation was performed as previously described by Yang et al. [29].

### siRNA transfection of OECs

To evaluate the involvement of IL-4 in microglial polarization, OECs at 1 × 10^5^ cells/cm^2^ grown in the 60 mm dishes were transfected with 0.5 μg of siRNAs (GenenPharma, Shanghai, China) targeting IL-4 using Lipofectamine® 2000 Transfection Reagent (Invitrogen, NY, USA) as recommended by the manufacturer. In brief, when confluence of OECs reached 85%, the formed complexes of Lipofectamine 2000 and IL-4–siRNA according to the manufacturer’s protocol were added in the cells. Forty-eight hours after transfection, cell supernatants and lysates were harvested to assess the efficiency of siRNA knockdown. The level of IL-4 mRNA was measured by quantitative RT‒PCR. The sequences are displayed in Supplementary Table 1. Meanwhile the level of IL-4 protein expression was quantified by western blot, and IL-4 levels were determined by ELISA (IL-4 ELISA kit; BD Biosciences, San Jose, CA) as described below. For transfection cytotoxicity, the viability of transfected cells was evaluated by trypan blue exclusion as described.

### M1 to M2 phenotypic conversion

To evaluate the impact of aOECs on M1 to M2 phenotype conversion, M1 microglial activation was first induced by the administration of 1 μg/ml LPS into microglial cultures. For M1 to M2 phenotype conversion, half of the medium was replaced with aOECCM following microglia treatment with LPS for different times (0 h, 6 h and 12 h), and the cultures were continuously maintained for 36 h. In parallel, IL-4 NTAb (10 ng/mL) was added into the microglial cultures in combination with half of the medium of RNAi-aOECCM and maintained for 36 h. Subsequently, cell lysates were prepared, or the cells were fixed with 4% paraformaldehyde for 30 min to examine the expression of M1 and M2 phenotype markers. All data are representative of at least three independent experiments.

### Quantitative real-time PCR

Total RNA was extracted from microglia undergoing the abovementioned treatments using RNAeasy (Qiagen), according to the manufacturer’s instructions. One microgram of total RNA was reverse transcribed into cDNA using the PrimeScript RT reagent kit (Takara). Ten nanograms of cDNA was used for PCR amplification. Quantitative RT‒PCR was performed in three replicates of each sample and repeated 3 times, and the reaction conditions were as follows: 94 °C for 5 min, 35 cycles at 94 °C for 30 s, 58 °C for 30 s, and 72 °C for 1 min. The genes of interest were CXCL1, TLR4, IL-1β, IL-6, CD86, iNOS, CD206, Arg-1, IL-10 and Ym-1. The mRNA levels were quantified by SYBR green-based quantitative real-time PCR (Takara) using an ABI Prism 7900 HT (Applied Biosystems, Foster City, CA, USA). The results were confirmed in at least 3 separate analyses. The qPCR primer sequences are listed in Supplementary Table 2. The housekeeping gene glyceraldehyde 3-phosphate dehydrogenase (GAPDH) was used as an internal control to normalize the PCR for the amount of RNA added to the reverse transcription reactions and the target gene expression was normalized to the control. Data are expressed as the fold change relative to the target gene/GAPDH.

### Western blot

The differently treated microglial cells were lysed in RIPA buffer supplemented with PMSF and phosphatase inhibitors for 30 min on ice, and crude extracts were made by passing the lysates through a 1 ml syringe with a 23 gauge needle and sonication to shear DNA. Protein samples were harvested by centrifugation at 12,000 rpm, and the concentrations of the clarified lysates were measured using a bicinchoninic acid kit. The supernatant was collected, and western blot were performed according to as a previously described protocol [45]. The following antibodies were used: PSR, TG2, Arg-1, CD86, iNOS, CD206, Arg-1, IL-10, C-Caspase3, β-tubulinIII, JAK1, p-JAK1, SOCS1, SOCS3, STAT6, p-STAT6, STAT1, p-STAT1, NF-κB65, p-NF-κB65, p-STAT3, and STAT3. All primary antibody dilutions were applied according to the manufacturer’s recommendations for titration or with minor adjustments. β-Actin was used as an internal control. After three washes with TBS containing 0.01% Tween 20, immunoblots were visualized with ECL detection reagent (Pierce). Densitometric analysis of bands was repeated three times and the integrated densitometry value (IDV) was calculated.

### Immunofluorescence

For immunostaining of each experimental group, all cultures on coverslips were fixed with 4% paraformaldehyde for 20 min and permeabilized with Triton X-100 in PBS for 10 min, followed by incubation with 2% BSA and 5% corresponding secondary antibody serum in PBS for 1 h at room temperature. Primary antibodies were incubated with the specimens overnight at 4℃ in 1% BSA in PBS. The following primary antibodies were used: goat anti-GFAP (1:1,000), rabbit anti-p75 (1:500), mouse anti-Tuj-1 (1:1,500), rabbit anti-Iba-I (1:1,500), mouse anti-iNOS (1:200), rabbit anti-Arg-1 (1:200), mouse anti-CD86 (1:200), and rabbit anti-CD206 (1:500). After the primary antibodies were removed and the samples were fully washed with PBS three times, the secondary antibodies (Alexa Fluor 488/594 anti-mouse IgG, anti-rabbit IgG, and anti-goat IgG) were diluted in PBS and incubated for 1 h in the dark at room temperature. The nuclei were counterstained with DAPI. Finally, the coverslips were mounted onto glass slides by using an anti-fading mounting medium. For evaluation of in vivo polarization of microglia undergoing different treatments, immunostaining of the spinal cord at 2 weeks after OEC transplantation was performed according to a previously protocol [28]. Finally, spinal cord sections were mounted onto glass slides for observation using confocal microscopy.

### Distinct treatments of neurons

To determine if the converted M2 phenotype microglia from M1 in aOECCM culture conditions were neuroprotective and enhanced neuron survival, spinal cord neurons cultured on coverslips or 6-Transwell/insert systems were divided as follows: 1) normal neurons cocultured with microglia; 2) normal neurons cocultured with microglia pre-exposed to LPS; 3) normal neurons cocultured with microglia preexposed to LPS supplemented with aOECCM; 4) normal neurons cocultured with microglia preexposed to LPS supplemented with aOECCM and IL-4 NTAb; 5) normal neurons cocultured with microglia exposed to IL-4 NTAb; 6) normal neurons treated with LPS, and 7) normal neurons only. Cells from all groups were maintained at 37 °C in a humidified 5% CO_2_ atmosphere for 3 days. Notably, microglial cells were seeded in inserts and pretreated with the LPS + aOECCM, LPS + aOECCM + IL-4 NTAb, LPS alone, or IL-4 NTAb or aOECCM alone for 12 h prior to coculture with neurons (in well). Concomitantly, cells were processed for following different examinations including Cell viability, Cleaved-Caspase-3, Flow cytometry, and neurite growth, respectively.

### Cell viability assay

Cell viability was assessed with the MTT assay using a 96-well culture plate, as previously described [28]. Briefly, neuronal cells were cocultured with LPS-treated microglial cells in the presence or absence of aOECCM and/or NTAb for 36 h. Notably, neuronal cells were seeded in the upper Transwell inserts, and microglial cells were seeded in the lower chambers of 24-well plates. Subsequently, MTT solution at 5 mg/mL was added directly to each well and incubated at 37 °C for 4 h. After incubation for 2 h at 37 °C, the upper inserts were transferred to another 24-well plates, and 300 mL dimethylsulfoxide was added to each well followed by incubation and shaking for 10 s to thoroughly dissolve the insoluble formazan. Finally, the solution in each well was transferred to three wells of a 96-well culture plate, respectively, and the absorbance at 570 nm was read on a microplate reader (Thermo Scientific).

### ELISA

To detect the efficacy of the siRNA-mediated IL-4 knockdown, an IL-4 ELISA Kit (Abcam, ab100747) was used according to the manufacturer's instructions to quantify the levels of IL-4 in the supernatant of aOECs. Optical density (OD) was measured at 450 nm using a microplate reader (Thermo Scientific).

### Flow cytometry

For examination of neuron survival under the different treatments mentioned-above, a PE Annexin V Apoptosis Detection Kit I (BD) was used to evaluate neuronal apoptosis by means of a FACS Canto II flow cytometry system (BD). All groups of neurons cocultured with microglial cells in Trans-wells were detached with 0.125% trypsin and 0.02% ethylenediaminetetraacetic acid (EDTA), washed and resuspended in cold PBS two times. A single-cell suspension was made by gentle triturating and filtering through 60 mm nylon mesh, and the final cell density was adjusted to 10^6^ cells/ml. Concomitantly, 100 μl of cell suspension was pipetted and mixed with 5 μl PE Annexin V and 7-AAD, and incubated at room temperature in the dark for 15 min. Prior to flow cytometry analysis, each sample was washed twice with PBS and resuspended in 400 μl of 1 × binding buffer. All samples were analyzed by cytometry within 30 min, and the results analysis was performed by using FlowJo software.

### SCI model and cell transplantation

To test the microglial polarization toward the M2 phenotype in vivo following transplantation of aOECs into the injured spinal cord, we performed development of an animal SCI model by weight-induced spinal contusion at T9-10, aOEC transplantation and histological staining study. In brief, 25 adult SD male rats weighing 250 g at an age of 2–2.5 months. were used as recipients and randomly divided into five groups. Five rats in each group were anesthetized intraperitoneally with 1% sodium Phenobarbital (50 mg/kg). When the anesthesia was successful, the rats were immobilized in the prone position and SCI animal model and cell transplantation were conducted according to our previously reported method [28]. Notably, all rats were maintained in identical housing conditions with a constant light cycle 12/12 h (light/dark), free access to water and food, and room temperature at 23–25 °C. In addition, all surgery procedures should comply with the above-mentioned animal Ethics and experimentation procedures. To investigate whether IL-4 mainly contributes to the microglial polarization toward the M2 phenotype, an implanted RNAi-aOEC suspension containing 10 ng/ml IL-4 NTAb was used for transplantation into the core site of the injured spinal cord. Notably, for other corresponding controls, the animal received the same volume of saline, IL-4 NTAb or aOECs. Among a randomized group of SD rats, a number of five SCI/four sham animals per group were used as recipients for each experiment. After injection, penicillin (10^4^ U) and gentamicin (8 × 10^4^ U) were subcutaneously administered to each rat for 3 days to prevent urinary tract infection. Moreover, the bladders of rats were manually and gently massaged 3 times every day to avoid retention of urine until the reflexive control of micturition was restored or the animals were sacrificed.

### Cell count and the assessment of neurite length

To quantify microglial polarization from the M1 to M2 phenotype or neuronal survival, cell counts were performed on five coverslips by observing 15 randomly selected fields of view per coverslip with a fluorescence microscope under 20 × magnification. Each observation field is approximately 0.45 mm^2^. M2 phenotype (double immunoreactivity for Arg-1 and Iba-1) or neuron counts were performed as previously described after cultures were terminated at each time point [43]. The cell numbers of at least five coverslips for each group were used for count analyses. Notably, to discriminate between the experimental and control groups, counting was performed in duplicate according to the same counting criterion by an unbiased, blinded investigator to the experiment. For the cell count of the slides, the criterion was the same as that for the cell coverslips. Notably, the inner boundary zone of the injury and the injection area of five animals were analyzed (Supplementary Fig. 2). As for quantitative neurite length, the assessment was performed as described previously [26, 29]

### Behavioral assessments

For evaluation of the efficacy of aOEC anti-inflammation by IL-4 modulating the M1 to M2 microglial phenotype switch, we conducted behavioral measures of the SCI rat model after cell transplantation as afore-mentioned. The behavioral tests, including Basso, Beattie, and Bresnahan (BBB) locomotor rating scale, rump-height index (RHI) assay, and the cylinder test, are relatively safe, easily performed. These behavioral measures were performed as described previously [24, 28, 46–48]. Notably, only rats with stable or prominent symptoms were used for behavioral tests, and assays were performed at six time-point (1, 3, 7, 10, 14, 21, and 28 days after SCI) post-transplantation. In addition, the functional locomotor assessment of five rats were performed for each of various groups.

### Statistical analysis

All data presented represent results from at least three independent experiments. For two group comparisons, statistical analysis was performed using Student’s t-test or the Kolmogorov–Smirnov test. Post hoc Tukey's analysis was applied for multiple group comparisons. Statistical significance was defined as Values of *P < 0.05.

## Results

### Characterization and identification of OECs, neurons and microglial cells

**Fig. 1:**
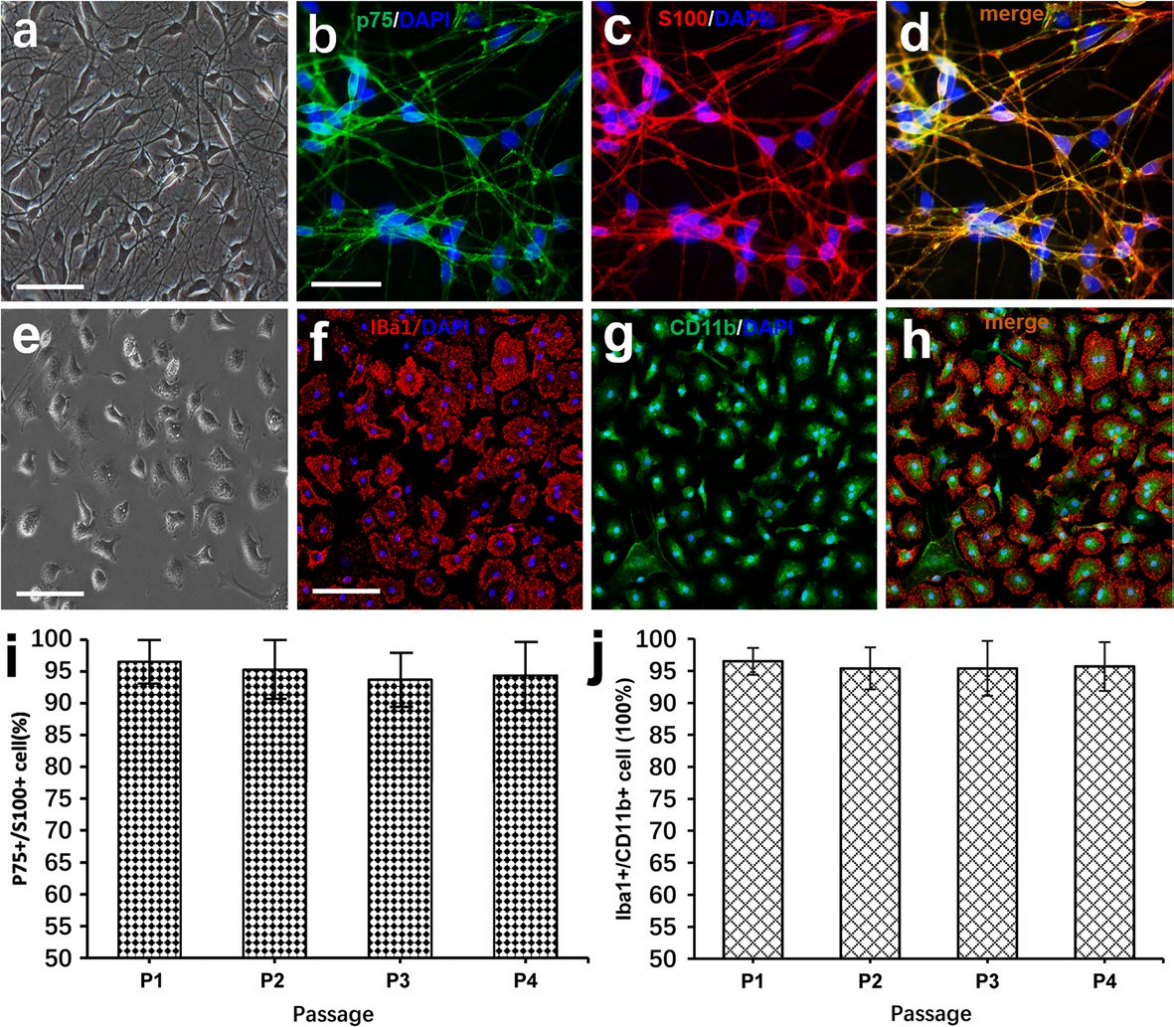
Morphological and biochemical characteristics of primary olfactory ensheathing cells (OECs) and microglial cells. **a** Phase-contrast microscopy showing morphology of primary OECs after purification at 5 days in vitro. **b**, **c** and **d** Double immunostaining with p75 and S100 in purified OECs, respectively. **c** Primary microglial cells at 3 days in vitro. **e**, **f** and **g** Representative photomicrographs of immunofluorescence for Iba1 and CD11b in purified microglial cells. **h** Percentages of p75^+^/S100^+^ cells among purified cells after sub-culture from passages 1–4. **i** Percentages of IBa1^+^/CD11b^+^ cells among purified cells after sub-culture from passages 1–4. The error bars indicate the SD of triplicate values. All data are reported as the means ± SEM. Scale bars, 100 mm

To investigate whether activated OECs can trigger the polarization of M1 to M2 microglial phenotype switch through the IL-4 signaling cascade, we first cultured and identified primary OB OECs and cortical microglia, respectively. Phase-contrast microscopy showed that almost all cells exhibited a flat and polygonal morphology with thin and long extensions, and formed an intricate network at 5 days after purification (Fig. 1a). To validate the identity of the purified cells, double immunostaining with P75 and S100 was subsequently conducted. Notably, these cells were positive for both P75 and S100, typical markers of OECs (Fig. 1b-d), suggesting that the purified OECs were not contaminated with other types of cells. For primary microglia, we found that after purification at 3 days in vitro, primary microglia entered the quiescent state and were of irregular and flat in shape with short extensions, and there were large amounts of granules filled in the cytoplasm, showing the special morphology profiles of glial cells (Fig. 1e). In addition, immunofluorescence staining demonstrated that these cells can express Iba-1 and CD11b, two specific markers for microglial cells (Fig. 1f–h). In agreement with the immunostaining observations, quantitative analysis demonstrated that more than 94.5% of cells were p75/S100 double-positive cells despite consecutive subculture for at least four passages (Fig. 1i). Likewise, the proportion of Iba-1^+^/CD11b^+^ double-positive cells accounted for over 95% of the total cells (Fig. 1j), and no significant difference was detected among the four passages, indicating that the cultured OECs and microglial cells could be used for subsequent experiments.

### Identification of activated OECs

**Fig. 2:**
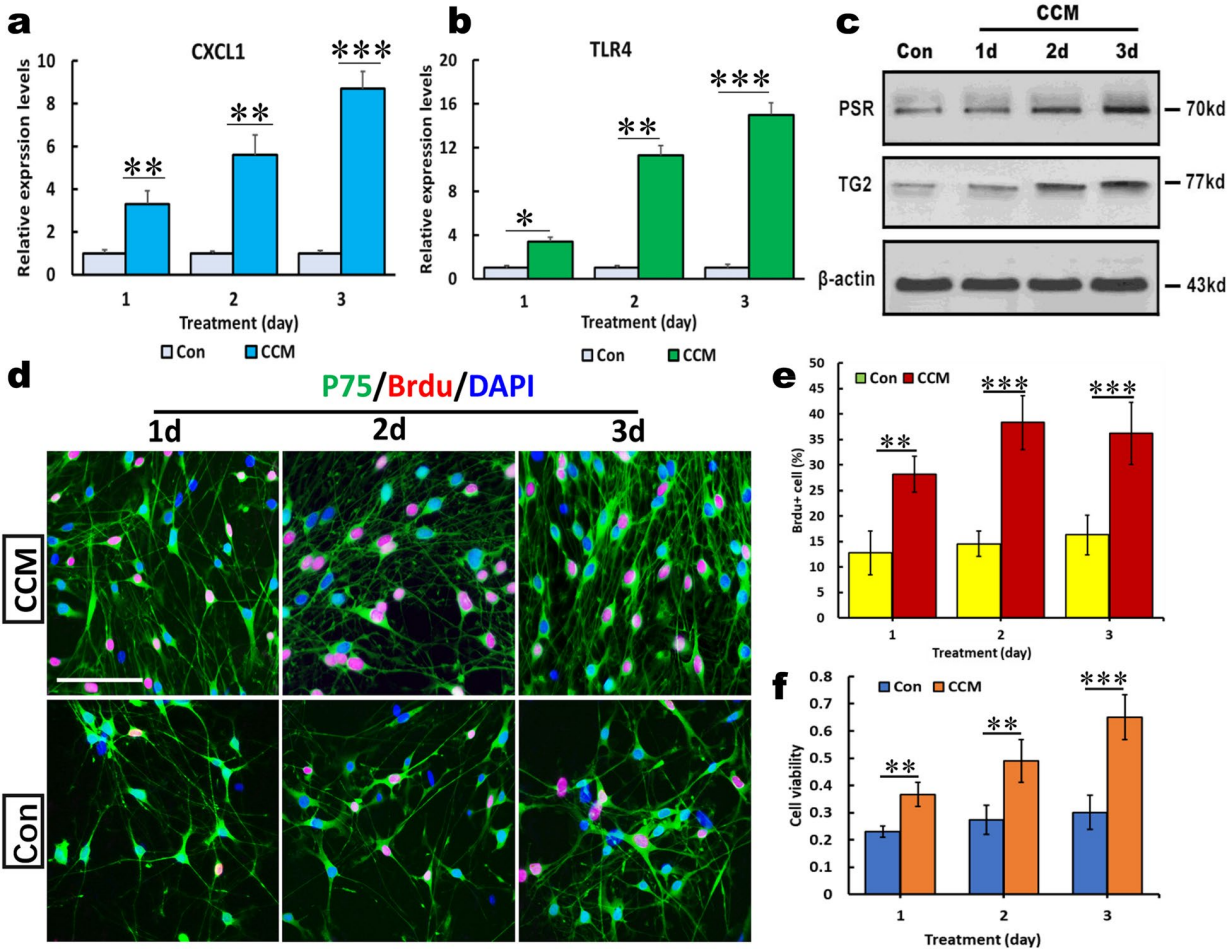
The effect of curcumin (CCM) on the activation of olfactory ensheathing cells. **a** and **b** Real-time polymerase chain reaction showed that mRNA levels of CXCL1 and Toll-like receptor 4 (TLR4) in OECs stimulated with CCM for 1, 2 and 3 days, respectively. **c** Western blot analysis of TG2 and PSR expression in OECs under the indicated conditions for 1, 2, and 3 days. β-actin served as a loading control of total proteins. **d** Representative photomicrographs of BrdU incorporation into p75-positive cells to assess OEC proliferative capacity after treatment with CCMs for 1, 2, and 3 days. **e** Quantification analysis of BrdU-positive OECs. **f** Quantification of the proliferation rate in MTT assays revealed that CCM significantly promoted OEC proliferation. OEC proliferation was time-dependent. All data are reported means ± SEM, and representative of 3 independent experiments with similar findings. **p < 0.01 and ***p < 0.001 vs controls (Con). Scale bars, 100 mm

To further confirm our hypothesis, we treated OECs with CCM for 1, 2, and 3 days, and identified the expression of several characteristic molecules crucial for OEC activation. As shown in Fig. 2a-c, after 1, 2 and 3 days of treatment with CCM, the mRNA levels of CXCL1 and TLR4 in OECs were drastically increased in a time-dependent manner, and a significant difference was found between the CCM-treated and untreated cells at the corresponding durations. Consistently, western blotting revealed a significant upregulation of PSR and TG2 expression, suggesting that the signaling pathway responsible for the activation of OECs was activated (Fig. 2c). In addition, the elevation of cell proliferation and viability, as crucial hallmarks of activated OECs, were measured and quantitated using BrdU incorporation and MTT assays. The results showed that the aforementioned treatment significantly increased the number of BrdU^+^ OECs, and quantification revealed a significant difference in the percentage of BrdU^+^ OECs between the 2 groups (Fig. 2d, e) (**p < 0.01 and ***p < 0.001 vs. the corresponding controls). Intriguingly, CCM significantly enhanced the viability of OECs compared with the untreated cells in the control groups (Fig. 2f). These data suggest that CCM can stimulate OEC activation.

### aOECs reverse the expression of LPS-induced M1-related genes and improve the expression of M2-related genes in microglia

**Fig. 3:**
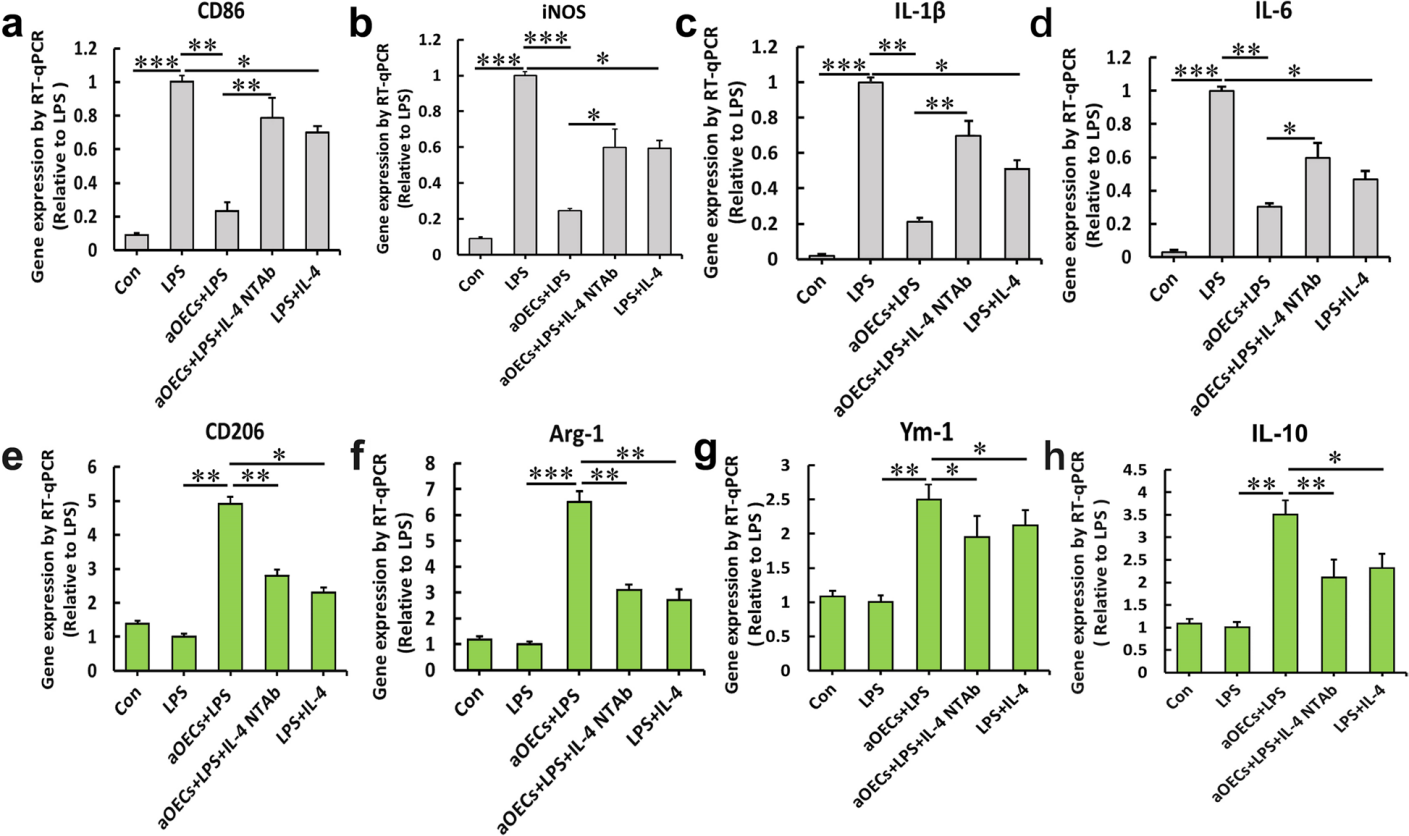
In vitro analysis of M1 and M2 polarization markers in microglial cells following exposure to proinflammatory stimuli and blockade of IL-4 signaling from aOECs. mRNA expression for M1 phenotypic genes CD86 (**a**), iNOS (**b**), IL-1β (**c**) and IL-6 (**d**) or M2 phenotypic genes CD206 (**e**), Arg-1 (**f**), YM-1 (**g**), IL-10 (**h**) using real time RT-PCR. Data are reported as means ± SEM of 3 independent experiments. *p < 0.05, **p < 0.01, and ***p < 0.001 compared with the corresponding controls

Our previous studies have demonstrated that transplantation of aOECs can effectively ameliorate SCI and improve outcomes, which is partially attributed to the suppressive effect of aOECs on inflammatory insult, leading to neural survival and axonal outgrowth. However, very little is known about whether aOECs can modulate microglial polarization toward anti-inflammatory M2 phenotypes. To clarify this issue, we first incubated the LPS-induced microglia with the conditioned medium from OECs and evaluated the expression levels of typical proinflammatory M1-related genes (including CD86, TNF-α, iNOS, IL-6 and IL-1β) and anti-inflammatory M2-related genes (including CD206, Arg-1, Ym-1, and IL-10). The results of quantitative PCR analysis showed that after 24 h of stimulation with LPS, the mRNA expression levels of the aforementioned M1-related genes significantly increased in microglial cells, while the administration of aOECs significantly suppressed this increase. Intriguingly, the addition of IL-4 NTAb markedly reversed the aOECs-induced decrease in the expression levels of M1 markers in LPS-induced microglial cells by aOECs. Notably, the presence of IL-4 also caused a significant decrease in the expression levels of the abovementioned M1 markers in LPS-induced microglial cells, but this decrease was somewhat less than that in aOECs (*p < 0.05, **p < 0.01, and ***p < 0.001, Fig. 3a-d). Conversely, aOECs drastically upregulated the expression of M2-related genes and promoted microglial polarization to the M2 phenotype in LPS-induced microglial cells, displaying an approximately 2.5- to sixfold upregulation compared with the expression of M2-related genes in the LPS control, while IL-4 NTAb effectively reversed the upregulation of M2-related genes by aOECs. Moreover, treatment with IL-4 also upregulated these M2-related genes, but the upregulation was still lower than that of aOEC induction (*p < 0.05, **p < 0.01, and ***p < 0.001, Fig. 3e-h). These data suggest that IL-4 released from aOECs is likely involved in modulating the M1 to M2 microglial phenotype switch.

### aOEC treatment shifted microglial M1/M2 polarization subsequent to LPS stimulation

**Fig. 4:**
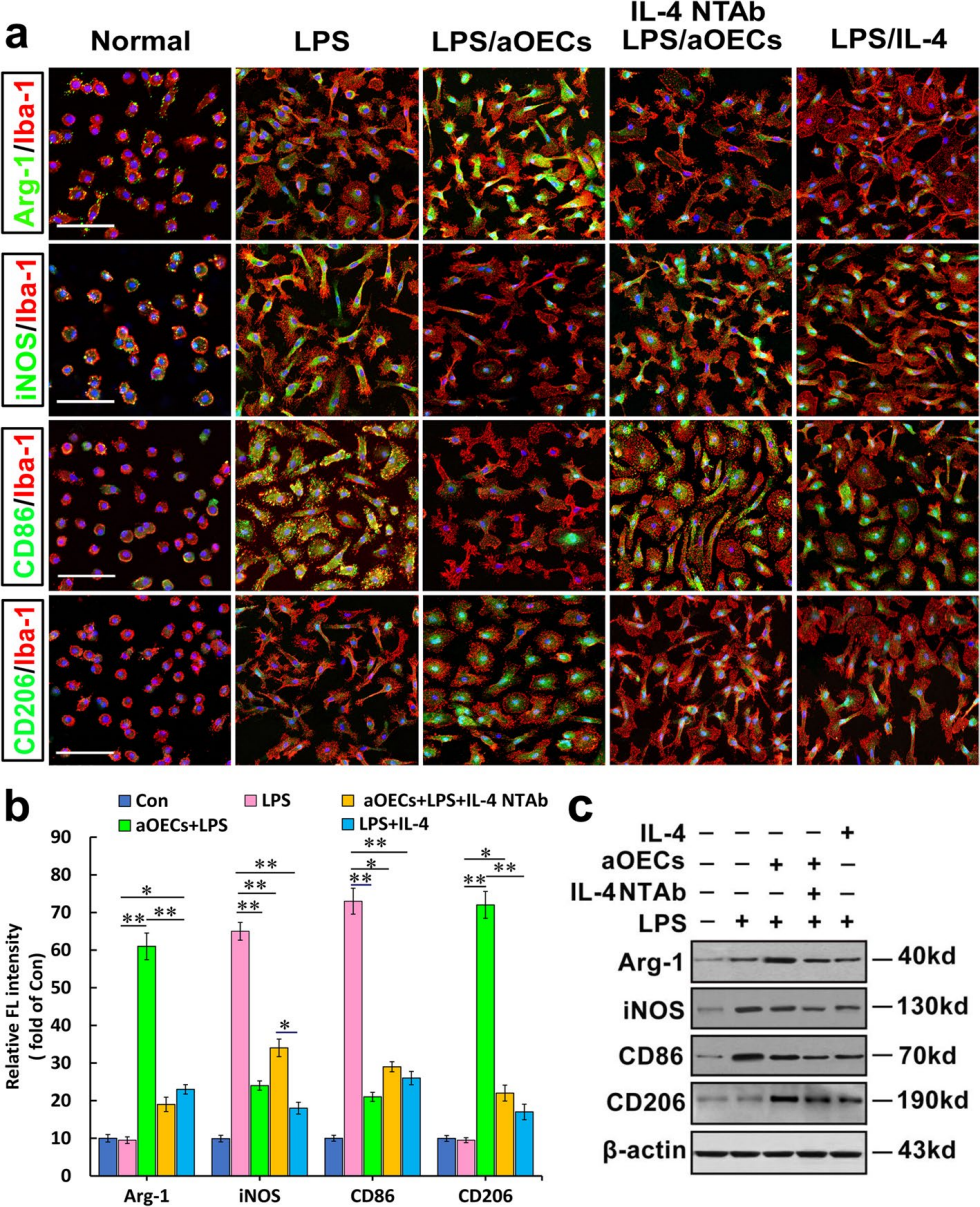
Immunohistochemical analysis of involvement of IL-4 from aOECs in microglial polarization from M1 to M2 switch in LPS-induced microglial cells. **a** Immunofluorescence revealed expression of M1 markers (iNOS and CD86) and M2 markers (Arg-1 and CD206), respectively, in Iba1-positive cells treated under the indicated conditions for 36 h. **b** Quantification of the cytosol intensity for the indicated M1 and M2 markers. **c** Western blot analysis of M1 and M2 marker expression in microglial cells treated with the indicated conditions. All data are shown as the mean ± SEM of 3 independent experiments and normalized to normal controls. *p < 0.05, **p < 0.01 vs corresponding controls (Con). Scale bars = 100 μm

To further validate the effect of aOECs on LPS-induced microglia polarization via modulation of IL-4, we used immunofluorescence to identify whether aOECs reduced the expression of phenotype M1 marker CD86 and pro-inflammation mediator iNOS, and increased the levels of phenotype M2 marker CD206 and Arg-1 expression in microscopic view. As shown in Fig. 4a, the expression of the M1 markers CD86 and iNOS, as well as the M2 markers Arg-1 and CD206, was undetectably low in Iba-1^+^ microglia under the normal culture. Meanwhile, most of the Iba-1^+^ microglia frequently showed a small and rounded shape, a typical feature of the resting microglia state (Fig. 4a leftmost column of panel). After LPS stimulation for 24 h, almost all Iba-1^+^ cells exhibited intense immunoreactivity for CD86 and iNOS, while no Arg-1 and CD206 reactivity was detectable in Iba-1^+^ cells (Fig. 4a second leftmost column of panel). Nevertheless, when LPS-induced microglia were incubated with aOECs, the intensity of immune-reactivity for CD86 and iNOS was remarkably attenuated and even appeared less reactive, which was significantly lower than that of the LPS-induced group. Instead, the immunoreactivity for Arg-1 and CD206 was significantly increased and higher than that of the LPS-induced and normal groups (Fig. 4a middle column of panel). Furthermore, the administration of IL-4 NTAb or siRNA-IL-4 in aOECs markedly reduced both Arg-1 and CD206 immunoreactivity, but reversely elevated CD86 and iNOS expression in LPS-stimulated microglia. Comparatively, both Arg-1 and CD206 immunoreactivity were more pronounced in the aOEC treatment group than in the aOEC plus IL-4 NTAb. In contrast, CD86 and iNOS immunoreactivity in the presence of IL-4 NTAb was completely opposite to what Arg-1 and CD206 expressed. A significant difference was detected between the 2 groups with or without IL-4. To assess whether IL-4 authentically promoted microglial polarization to the M2 phenotype in LPS-stimulated microglial cells, we treated microglial cells with IL-4. Our results showed that IL-4 markedly suppressed the expression of CD86 and iNOS in LPS-induced microglial cells and markedly increased their expression. In comparison, the decreased expression of M1 markers and increased expression of M1 markers in LPS-induced microglial cells caused by IL-4 were slightly lower than those caused by aOECs. To verify the effect of IL-4 on microglia M1/M2 polarization switch, the intensity of the aforementioned M1 and M2 marker colocalization with Iba-1 was further assessed. Quantification showed that there were significant differences in the intensity of these markers between the indicated groups (*P < 0.05, **P < 0.01, Fig. 4b). In addition, the western-blot analysis showed that aOECs could elicit the polarization of LPS-induced M1 microglia toward M2, displaying higher levels of Arg-1 and CD206 and relatively lower levels of CD86 and iNOS in LPS-induced microglia. Strikingly, inhibition of IL-4 in aOECs by IL-4 NTAb and siRNA significantly attenuated the decrease in CD86 and iNOS levels, and increased Arg-1 and CD206 levels by aOECs (Fig. 4c), suggesting that IL-4 released by aOECs likely plays a critical role in modulating the M1 to M2 microglial phenotype switch.

### aOECs regulated the polarization of microglia from the M1 to M2 phenotype in the lesion area after SCI

**Fig. 5:**
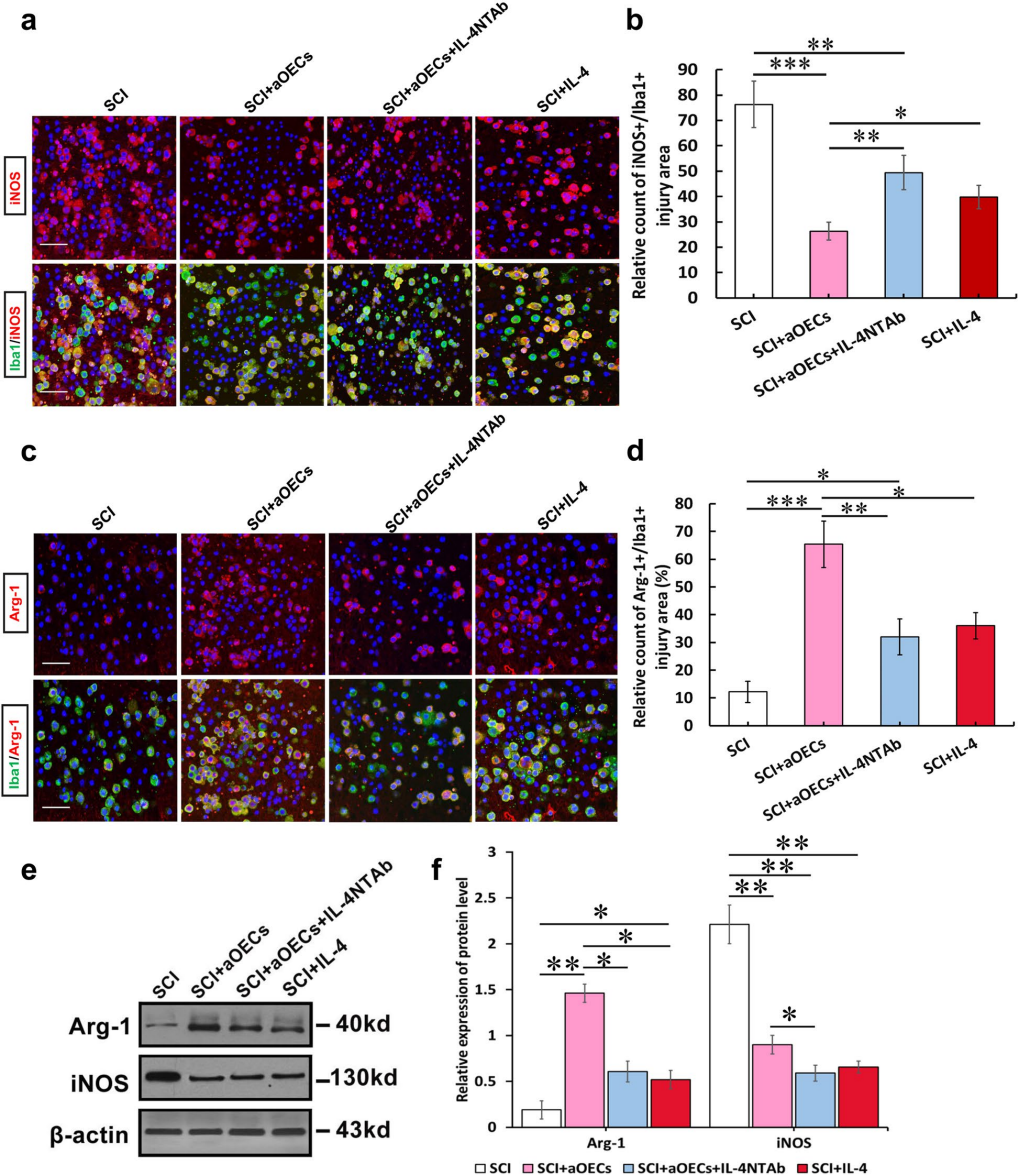
IL-4 from aOECs increased microglial anti-inflammatory polarization and reduced the pro-inflammatory cells after SCI. **a** Representative images of immunostaining of iNOS and Iba1 in saline, OECs, IL-4, or IL-4-si OECs-treated rats at 14 dpi. Scale bar = 100 μm. **b** Quantification of iNOS-positive microglia in the bilateral areas rostral and caudal to the lesion site. **c** Immunostaining of Arg-1-positive cells in saline, OECs, IL-4, or IL-4-si OECs-treated rats at 14 dpi. Scale bar = 100 μm. **d** Quantification of Arg-1-positive microglia in the bilateral areas rostral and caudal to the lesion site. Note that aOEC treatment increased the numbers of Arg-1-positive microglia/macrophages, the effect was significantly weakened by IL-4-si and NTAb *n* = 5/group. **e** Western blot analysis of Arg-1 and iNOS expression in the injury area at 14 dpi under the indicated treatments. **f** Quantification of expression levels of Arg-1 and iNOS normalized to β-actin. *n* = 10, *p < 0.05, **p < 0.01 ***p < 0.001

To determine whether aOECs exert modulatory effects similar to those observed in vitro, aOECs or IL4-silenced aOECs (Supplementary Fig. 1) with IL-4 NTAb were injected into injured rats to determine the effects of aOECs on the polarization of microglia after SCI. Fourteen days after cell transplantation, we evaluated the characteristic polarization of microglia in the lesion areas of different groups using the representative M1-associated marker iNOS and M2-associated marker Arg1 for double immunofluorescence staining together with Iba-1, which detects microglia/macrophages in the spinal cord. Similar to the in vitro immunofluorescent data, immunofluorescence staining showed that the number of iNOS-positive microglia/ macrophages was significantly decreased upon transplantation in the aOEC group compared with the SCI group; however, IL-4-silenced aOECs with IL-4 NTAb significantly reversed this decrease. Notably, the delivery of IL-4 also partially reduced the expression level of iNOS, but the reduction was still lower than that of induced by aOECs (*n* = 5, *p < 0.05, **p < 0.01, and ***p < 0.001 Fig. 5a, b). In addition, we examined the effect of aOECs on anti-inflammatory polarization. Our data further revealed that the highest level of Arg-1 in the microglia/macrophages was observed in the lesion areas of the aOEC-transplanted group compared with the SCI and other treatment groups. Likewise, the delivery of IL-4 also increased the number of Arg-1-positive microglia/macrophages, but the increase was lower than that of aOEC-induced microglia/macrophages (*n* = 5, *p < 0.05, **p < 0.01, and ***p < 0.001 Fig. 5c, d). In line with the immunofluorescence results, the western-blot analysis further showed that aOECs upregulated the expression of the M2-related marker Arg1 and downregulated the M1-related marker iNOS (Fig. 4e, f), and the activity was intimately associated with IL-4 released from aOECs, which was again confirmed the above in vitro results (Fig. 4a-c). Consequently, these results demonstrate that aOECs could play a robust role in modulating neuroinflammation by releasing IL-4, which triggers the switch of microglia/macrophages from the M1 to the M2 phenotype.

### aOECs counteract LPS-induced inflammatory insults to spinal cord neurons

**Fig. 6:**
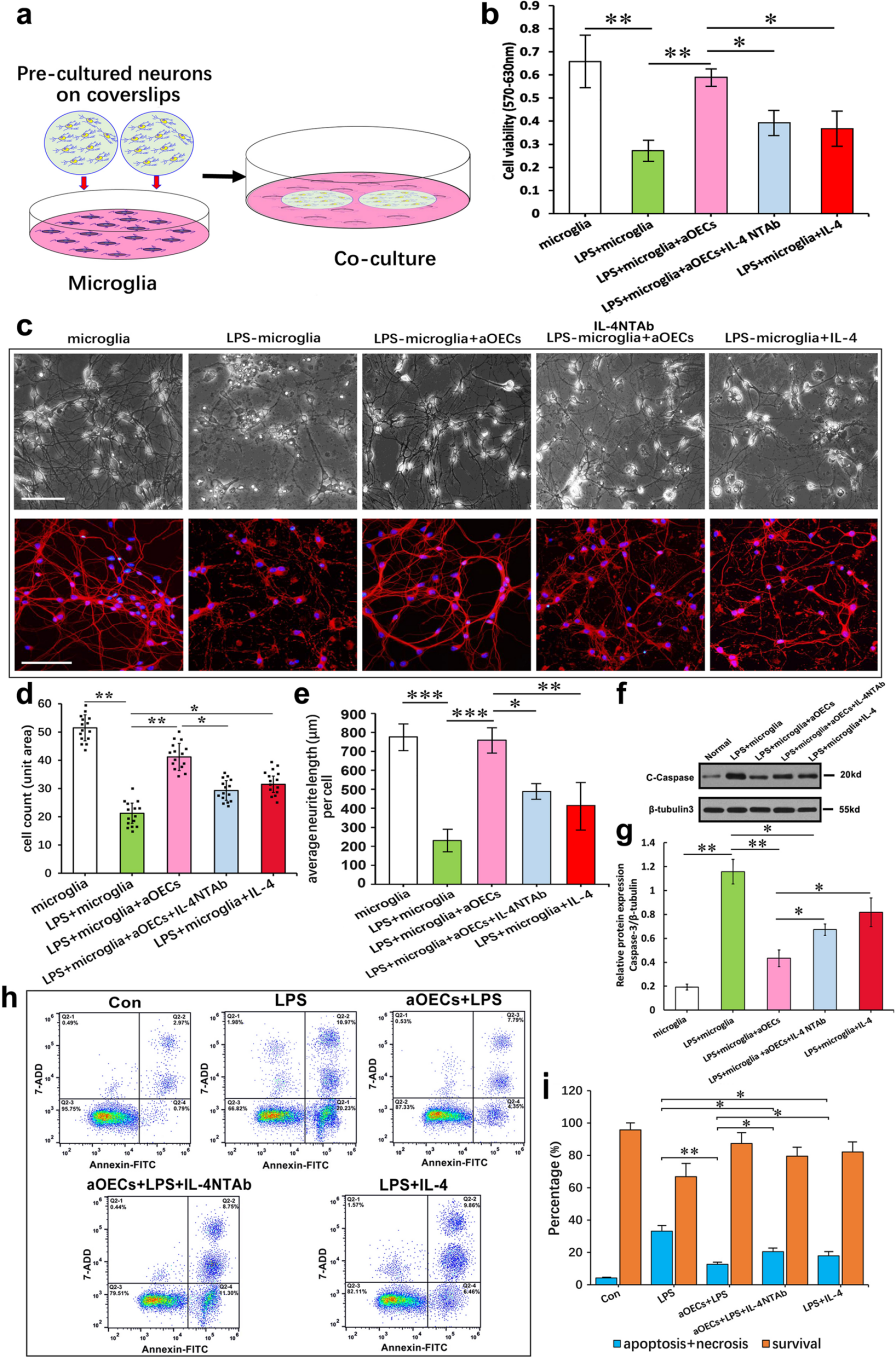
aOECs counteract LPS-induced pro-inflammatory insult of microglia to spinal cord neurons. **a** Schematic diagram showing co-culture of neurons with microglia treated with aOECs and other stimuli, respectively. **b** The viability of neurons co-cultured with microglia undergoing the indicated treatments, respectively. **c** Representative photographs of neurons co-cultured with LPS-treated microglia in the presence or absence of aOECs plus/minus IL-4 NTAb, respectively. **d** Quantitative assessment of the number of β-tubulinIII positive cells. Note that aOECs significantly suppressed the decreased number of β-tubulinIII positive cells by pro-inflammatory insult of microglia. Reversely, Blockade of IL-4 signaling by IL-4 NTAb results in a decrease of β-tubulinIII positive cells. **e** Quantitative assessment of neurite length under indicated treatments. **p < 0.05, **p < 0.01, and ***p < 0.001 vs their respective control, analysis of variance post hoc test. **f** Western blot analysis of C-caspase-3 expression in neurons under the indicated treatments. **g** Quantification of expression levels of C-caspase-3 normalized to β-tubulinIII. **h** The analysis of the apoptotic and viable neurons co-cultured with microglial cells undergoing the indicated treatments via flow cytometry. Notably, four quadrants represent the percentage of live, apoptotic and necrotic cells undergoing the indicated treatments, respectively. **i** Quantification of the relative number of apoptotic and necritic neurons co-cultured with microglia undergoing the indicated treatments. *n* = 3, *p < 0.05 and **p < 0.01 vs their respective controls. Scale bar = 100 μm

Our previous study and other reports have revealed that the microglial proinflammatory phenotype is tightly associated with neuronal degeneration and death after SCI, and the neuroinflammation usually impairs neuronal functions, thus determining the severity of SCI to a large extent. To further characterize the functional state of M2 microglia after the switch of microglial polarization by OEC-released IL-4, we cocultured spinal cord neurons with LPS-induced microglial in a medium containing aOECs or IL4-silenced aOECs with IL-4 NTAb, and evaluated several parameters indicative of neuronal survival and growth (Fig. 6a). As shown in Fig. 6b, aOECs significantly reversed the decrease in neuronal viability resulting from LPS-induced microglial inflammatory insult, while the administration of IL-4 NTAb remarkably attenuated the neuroprotection of aOECs against the inflammatory insult, displaying a significant decrease in cell viability. Although IL-4 alone also attenuated the decrease in cell viability caused by inflammatory insult, the suppression was weaker than that by aOECs. Moreover, neuronal growth following the abovementioned treatments was identified using microscopy and immunofluorescence. The results showed that almost all neurons grew well under normal culture conditions, exhibiting abundant, long, thin and branched processes with considerable arborization. However, those neurons cocultured with LPS-induced microglia, showed marked morphological distortions characterized by cell shrinkage, degeneration, numerous disintegrated neurites, and abundant scattered necrotic debris. In addition, the number of viable cells was significantly reduced. Strikingly, the addition of aOECs markedly ameliorated the LPS-induced morphological distortions and neurite disintegration. In contrast, IL4-silenced aOECs or the inclusion of IL-4 NTAb apparently weakened the neuroprotection of aOECs (Fig. 6c upper row of panel indicated by IL4-silenced aOECs). Similar to the microscopic view, Tuj-1 immunofluorescence analysis revealed that most Tuj1^+^ cells exhibited characteristically exuberant, with pronounced neurites and enhanced arborizations, and a strong fluorescent signal. No scattered degenerated debris or fragmented neurites were observed under normal culture conditions (Fig. 6c lower row of panel indicated by normal). However, Tuj1-positive cells cocultured with LPS-induced microglia exhibited a characteristic apoptotic morphology, displaying somatic shrinkage with severely disintegrated neurites. More strikingly, in addition to the more significant decline in the number of Tuj1^+^ cells, there was a large amount of cell debris scattered around the cells (Fig. 6c lower row of panel indicated by LPS). When cells were pretreated with aOECs for 4 h, the neuroinflammation insult induced by LPS-treated microglia was greatly attenuated, and the number of Tuj1^+^ cells with good suitable morphology was significantly increased. However, IL4-silenced aOECs or the inclusion of IL-4 NTAb markedly dampened the neuroprotective effect of aOECs on neurons (Fig. 6c lower row of panel indicated by IL-4 NTAb). Similar to aOECs, IL-4 also alleviated the insult by induced LPS-treated microglia, but its activity was weaker than that of aOECs (Fig. 6c lower row of panel indicated by IL-4). Furthermore, quantitative analysis of Tuj1^+^ cells indicated that reversed the neuronal death by LPS-induced microglia, as demonstrated by a significantly higher percentage of Tuj1^+^ cells (41.18 ± 4.8%). When the IL4-silenced aOECs or inclusion of IL-4 NTAb were administered in the coculture, the percentage of Tuj-1^+^ cells significantly decreased (29.31 ± 3.5%). When the addition of IL-4 alone was added, the percentage was 31.52 ± 2.9%, higher than that of LPS-induced microglia but lower than that of aOEC-treated microglia (*P < 0.05 and **P < 0.01 Fig. 6d). In addition, quantitative analysis of neurite outgrowth showed that LPS-induced microglia significantly inhibited the neurite extension, and the inhibitory effect can be reversed by aOEC conditioned media. Noteworthy, IL4-silenced aOECs or inclusion of IL-4 NTAb markedly attenuated the neurite growth-promoting effects of aOECs (*P < 0.05, **P < 0.01, and ***P < 0.001 Fig. 6e). These data suggest that aOECs confer neuroprotective effect against inflammation-induced insults by releasing IL-4 to modulate switching microglial polarization.

To further validate whether aOECs are involved in protecting neurons against LPS-induced neuroinflammatory insults through IL-4-evoked the switch of microglial M1/M2 polarization, we evaluated the expression of the apoptosis-related protein cleaved Caspase-3 in the neurons cocultured with LPS-induced microglia. As shown in Fig. 6f and g, LPS-induced microglia markedly upregulated cleaved Caspase-3 in the cocultured neurons compared with normal microglia, while this upregulation was dramatically reduced by more than 60% in the presence of aOECs. More importantly, treatment with IL-4 NTAb greatly attenuated cleaved Caspase-3 downregulation, while IL-4 alone effectively downregulated the expression of Caspase-3 (*n* = 3, *p < 0.05 and **p < 0.01), further indicating the neuroprotective events of aOECs against LPS-induced neuroinflammatory insults through IL-4 modulation. In addition, we examined the apoptosis and viability of neurons co-cultured with microglial cells undergoing the abovementioned treatments via flow cytometry. As shown in Fig. 6h and i, the percentage of apoptotic cells was markedly reduced by aOEC pretreatment (absolutely live, apoptotic and necrotic neurons accounted for 87.32%, 12.14%, and 0.53%, respectively), compared with that of the LPS group (absolutely live, apoptotic and necrotic neurons accounted for 66.82%, 21.20%, and 1.98%, respectively). In contrast, treatment with IL-4 NTAb apparently altered the absolute percentages of the cultured neurons with LPS-induced microglia pretreated with aOECs (absolutely live, apoptotic and necrotic neurons accounted for 79.51%, 20.05%, and 0.44%, respectively). Notably, the percentage of apoptotic neurons was significantly reduced, but the percentage of necrotic cells somewhat remained high in the LPS-induced microglia pretreated with IL-4. The absolute percentages of live, apoptotic and necrotic neurons were as follows: 82.11%, 16.32%, and 1.57%, respectively. The subsequent quantification of live cells revealed that aOECs markedly reversed neuronal apoptosis and necrosis induced by neuroinflammatory insult. These results substantiate the specificity of IL-4 from aOECs in switching microglial polarization toward a beneficial anti-inflammatory M2 phenotype as the cause of neuronal survival in our experiments.

### The crosstalk between JAK1/STAT1/3/6 signaling pathways and NF-κB/SOCS1/SOCS3 cascades orchestrated IL-4-mediated M2 polarization of microglia

**Fig. 7:**
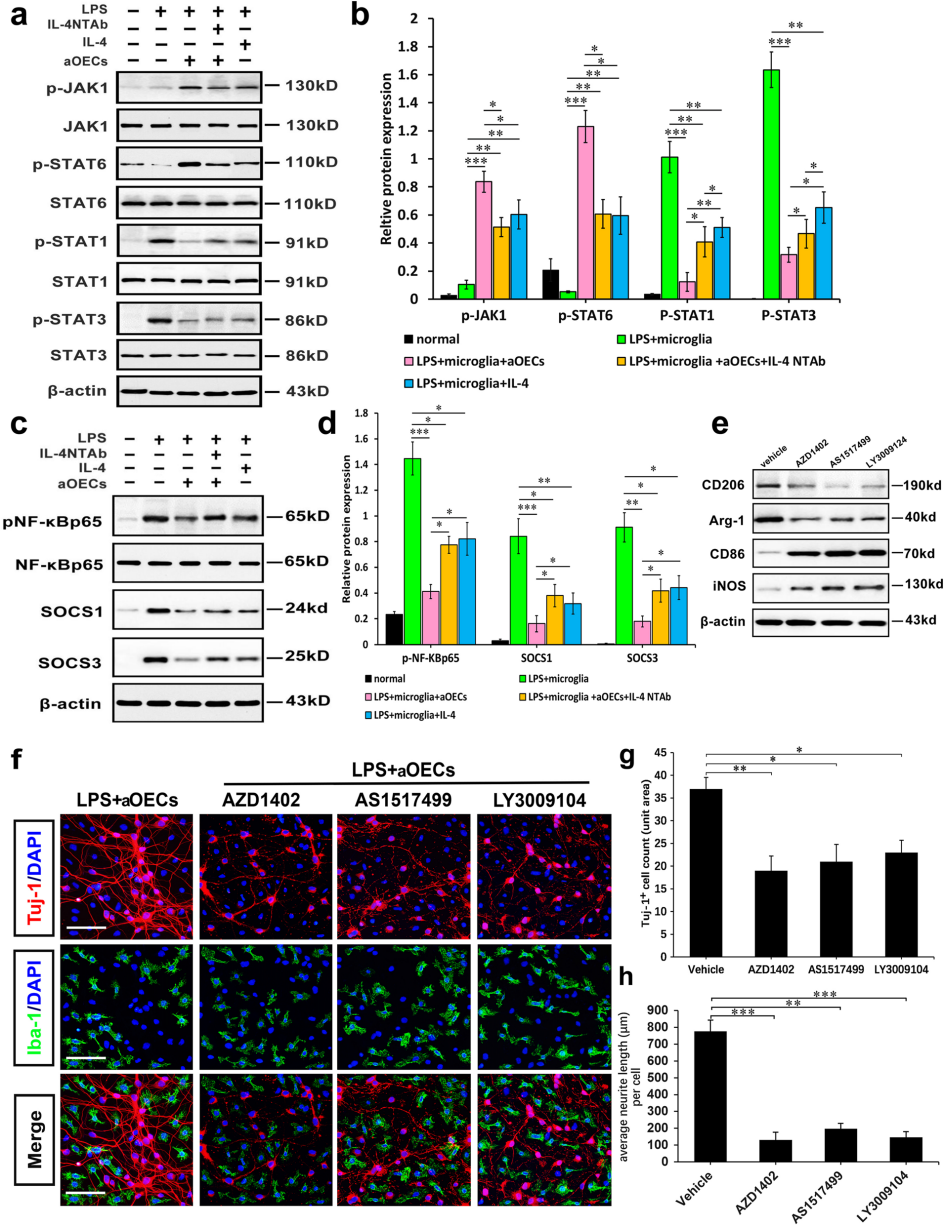
aOECs reversed neuroinflammation through crosstalk of JAK1/STAT1/3/6 signaling pathways and NF-κB/SOCS1/SOCS3 cascades to orchestrate IL-4-mediated M2 polarization of microglia. **a** Representative western blot band showed the levels of total protein (JAK1, STAT1, STAT3, and STAT6) and phosphorylated protein for the above-mentioned molecules in microglial cells under indicated stimulation for 24 h. **b** Quantitative analysis of the levels of phosphorylated protein (JAK1, STAT1, STAT3, and STAT6) in a, which were quantified and normalized to their non- phosphorylation. (*n* = 3/group). **c** Representative western blot band showed the levels of NF-κB, SOCS1, SOCS3, and phosphorylated NF-κB expression in microglial cells under the indicated conditions. β-actin served as a loading control. **d** Quantitative analysis of the levels of phosphorylated NF-κB, SOCS1, and SOCS3 (*n* = 3/group). All data are presented as means ± SEM. *P < 0.05, **p < 0.01, and ***p < 0.001. **e** Western blot analysis of M1 and M2 marker expression in microglial cells treated with the indicated treatments (several selective antagonists AZD1402 for IL-4Rα, AS1517499 for STAT6, and LY3009104 for JAK1). **f** The representative photographs of neurons co-cultured with microglia in the indicated conditions. Note that microglia pre-treated with several selective antagonists significantly inhibited the neuronal survival and neurite outgrowth. Scale bar = 100 μm. **g** Quantitative assessment of the number of Tuj-1^+^ cells co-cultured with microglia treated with antagonist as indicated, respectively. **h** Quantitative assessment of average length of neurite under indicated treatments. *p < 0.05, **p < 0.01, and ***p < 0.001 vs the corresponding controls

To gain novel insights into the molecular mechanisms underlying the phenotypic switch conversion of M1 toward M2 microglia, we investigated the involved signaling pathways and microglial M1/M2 phenotypic switching induced by IL-4 or other synergistic cytokines derived from aOECs. Given the pivotal roles of JAK-STAT signaling in skewing microglia toward either an M1 or an M2 phenotype, we first examined the protein levels of JAK1, p-JAK1, STAT6 and p-STAT6, STAT1 and p-STAT1, and STAT3 and p-STAT3 in the microglial cells following the abovementioned treatments by using western blot. The blotting results showed that the protein levels of p-STAT1/3 were significantly increased under LPS stimulation, while the levels of p-JAK1 and p-STAT6 dramatically decreased (Fig. 7 Lane 2). In the presence of aOECs, the levels of p-STAT1/3 were significantly decreased under LPS stimulation, and the levels of p-JAK1 and p-STAT6 were obviously elevated (Fig. 7a Lane 3). Strikingly, the treatment with IL-4 NTAb or IL4-silenced aOECs abrogated the increase in the protein levels of p-JAK1 and p-STAT6, and the downregulation p-STAT1/3, caused by aOECs (Fig. 7a Lane 4). Similar to aOECs, IL-4 alone also potently inhibited the LPS-induced activation of STAT1/3 abovementioned signal molecule expression, albeit being less potently than aOECs (Fig. 7a Lane 5). Quantitative analysis intuitively showed that there were significant differences in these protein levels between these groups (*P < 0.05, **P < 0.01, and ***P < 0.001 Fig. 7b). These data implied that IL-4 modulates the JAK1/1/3/STAT6 pathway to promote microglial polarization.

SOCS proteins act as cytokine feedback inhibitors by targeting the JAK/STAT pathway, and they are involved in microglial polarization and regulate inflammatory responses in microglia [49, 50]. More importantly, SOCS proteins, especially SOCS1 and SOCS3, are closely related to STAT1/3 activation and reported to participate in the transcriptional regulation of M1 polarization. In addition, the levels of SOCS3 modestly regulated LPS-induced the activation of NF-kBp65, which interacts with the DNA activating site responsible for M1 polarization. Therefore, the protein levels of SOCS and pNF-kBp65 were investigated to elucidate their role in orchestrating the microglial polarization. As shown in Fig. 7c and d, LPS apparently upregulated the expression of p-NF-κBP65, SOCS1 and SOCS3 in microglia, while aOEC pretreatment remarkably abrogated the LPS-induced upregulation of these molecules (Fig. 7c Lane 3 and 7d). Conversely, inclusion of IL-4 NTAb severely curtailed the effect of aOECs on LPS-induced p-NF-κBP65, SOCS1 and SOCS3 upregulation (Fig. 7c Lane 4 and d). Similar changes were observed following the direct administration of IL-4, and the above molecules were remarkably downregulated, with weaker potency, relative to that of aOECs (Fig. 7c Lane 5 and d). Based on these results, we speculate that IL-4 may predominantly modulate the M1/M2 polarization of glial cells through regulation of the NF-κB and SOCS1/3 signaling.

To further substantiate the involvement of JAK1/STAT1/3/6 signaling pathways and NF-κB/SOCS1/SOCS3 cascades in aOEC-mediated M1/M2 polarization events, microglia were pretreated with several selective antagonists, AZD1402 for IL-4Rα, AS1517499 for STAT6, and LY3009104 for JAK1, respectively, prior to aOEC and LPS stimulation. Western blot results showed that compared with aOECs alone treatment (vehicle), AZD1402, AS1517499, and LY3009104 significantly decreased the expression levels of CD206 and Arg-1, two typical markers for the M2 phenotype, and remarkably elevated the expression levels of CD86 and iNOS, two typical markers for the M1 phenotype after 2 days of treatment (Fig. 7e). These results suggest that the JAK1/STAT1/3/6 signaling pathways and NF-κB/SOCS1/SOCS3 cascades participate in the aOEC-mediated switch of microglial polarization. Nonetheless, it is still not clear whether the functional state of microglia polarization through the abovementioned signaling pathways, could affect neuron outgrowth. Consequently, we further assessed neuronal survival and neurite outgrowth by coculture with microglia treated with the abovementioned several selective antagonists, respectively, in the presence of aOECs and LPS. The results indicated that in OEC-pretreated microglia exposed to LPS, almost all Tuj-1-positive cells exhibited intact cell somas with long and thick neurites with excessive branching that formed an intricate network, while the inclusion of several selective antagonists resulted in apparent cell morphological distortion characterized by cell shrinkage, cytoplasmic injury and neurite fragmentation. More strikingly, there was extensive disintegration of neurites and a large amount of cell debris scattered in the cultures (Fig. 7f). Moreover, we quantified neuronal survival and neurite outgrowth. As shown in Fig. 7g and h, three selective antagonists dramatically suppressed neuron survival and neurite outgrowth when cocultured with LPS-induced microglia in the presence of aOECs, as indicated by significant reductions in both the number of surviving cells and average length of neurite when compared with controls (*p < 0.05, **p < 0.01, and ***p < 0.001), suggesting that the abovementioned signaling pathways responsible for switching microglial M1 to M2 were likely inhibited. The data further validated that aOEC-released IL-4 elicits microglial M1/M2 phenotypic switch through the JAK1/STATs and NF-κB/SOCS signaling pathways.

### aOECs improved sensorimotor function recovery after SCI

**Fig. 8:**
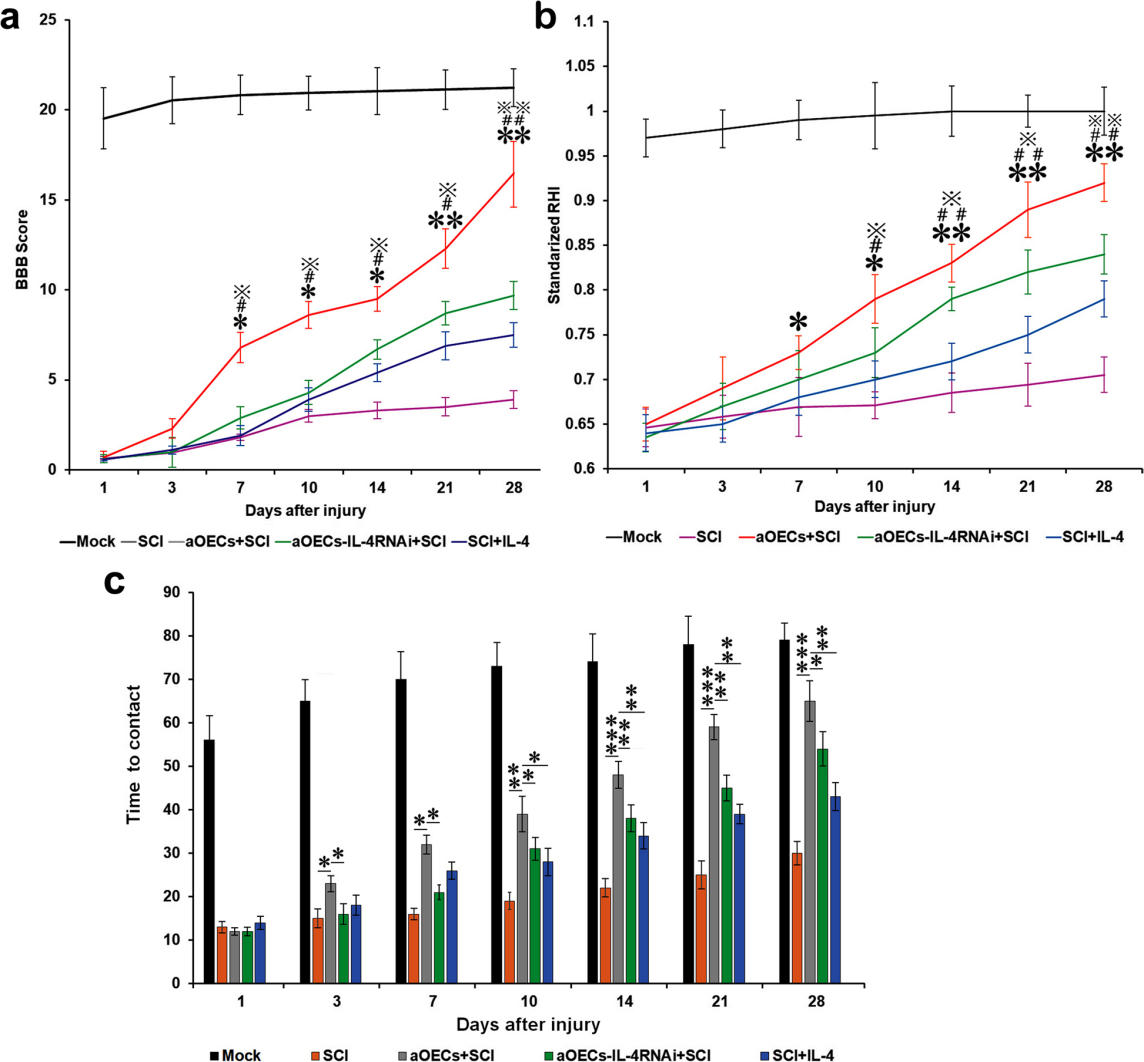
Transplantation of aOECs improved the neurological functions in rat models of SCI. **a** BBB scores within the different observation periods in distinct treated rats after compression injury of spinal cord. **b** RHI values at different time points after SCI. **c** Contact time in mock, aOEC transplantation, OEC-IL-4-RNAi transplantation, and IL-4 delivery rats at the indicative time post-surgery. Note that BBB scores and RHI values in distinct treated rats *n* = 5/group. *P/^#^P/^※^*P* < 0.05, **P/^##^P/^※※^*P* < 0.01, and ***P < 0.001 represent aOEC-transplanted rats compared with SCI (*), OEC-IL-4-RNAi transplantation + SCI (#), and IL-4 delivery + SCI (※) rats at the indicative time post-surgery, respectively

To further validate whether aOEC implants effectively improved sensorimotor behaviors in rats following SCI through IL-4 mediating M1 to M2 microglial phenotype switch. Behavioral measurements following aOEC transplantation were conducted. As shown in Fig. 8a, aOEC implants significantly improved the BBB score, and the score showed a progressive increase at five time-point in all animals following aOEC implantation. By comparison, the rats in aOEC transplantation group globally achieved a significantly higher level of post-injury motor recovery than that in other treatment groups (*P < 0.01, or **P < 0.05, compared at the same time point). Consistent with the BBB locomotor scale, the rump-height index of aOEC-transplanted rats showed a similar tendency to BBB score at the corresponding time points, and higher than that of other treatment groups (Fig. 8b). Likewise, greater frequency in the placement of two palms on the cylinder wall was found in the aOEC-engrafted rats when compared with the other treated rats (*P* < 0.05, Fig. 8c).

## Discussion

Spinal cord injury (SCI) is a largely irreversible and devastating neurological disorder with high mortality and disability [51]. Due to the complex and multifaceted mechanism of SCI pathology, no curative therapeutic strategies are currently available for SCI. Among the complex pathogenesis processes of SCI, excessive inflammatory responses, as the principle culprits in initiating and exacerbating all types of damage, are an important and inevitable pathological process and can largely contribute to the severity of SCI and the occurrence of neurological deterioration outcomes and even various uncontrollable sequelae [9, 52–56]. As a result, the urgent need for a clinically available approach to antagonize inflammatory responses may be of vital importance. Fortunately, emerging evidence has revealed that OECs, a specialized type of glial cell, have increasingly become excellent cell-based therapy candidates for SCI. This potential is mainly attributed to the unique cellular characteristics of OECs, such as neurotrophic support, anti-neuroinflammation and immunomodulation [23–25, 27, 28, 42]. Our latest studies demonstrated that transplantation of aOECs promotes neural regeneration and functional recovery following SCI, and potentiates the neuronal differentiation of neural stem cells in the inflammatory environment [24, 28, 31]. Alternatively, our group also found that OECs efficiently inhibited the proinflammatory polarization of macrophage/microglia while enhancing the number of anti-inflammatory phenotype of microglia, resulting in neural function recovery after SCI [24, 28]. Nevertheless, the signaling molecules released from aOECs that exactly mediate microglial polarization to the anti-inflammatory M2 phenotype in the complicated cell events remains to be elucidated.

In this study, we present data emphasizing the potential efficacy of IL-4, as a critical driver, released from aOECs triggers the M1 to M2 phenotype transformation in microglia, resulting in an increased anti-inflammatory activity. Our results demonstrated convincingly that the OECs activated by CCM acquired enhanced proliferative capacity and the ability to secrete cytokines. In line with our recent work, we found that the upregulation of TG2 and PSR was involved in the activated OECs, resulting in biofunctional amplification. Accordingly, the enhanced potential of OECs to create a neuroprotective environment could be a promising option for the effective treatment of SCI and neurodegenerative diseases. Based on our more recent studies regarding anti-inflammatory prevention trials in SCI using aOECs, we found that the aOEC-mediated alleviation of inflammation is mainly attributed to its promoting M2 microglial polarization in addition to the secretion of anti-inflammatory factors, thus reducing the inflammatory insult in SCI [24, 28]. However, it is unknown how aOECs modulate M1/M2 polarization and the mechanism by which aOECs regulate microglial polarization has not been clearly elucidated. In our study, we first observed that aOECs remarkably inhibited the expression of M1 markers and cytokines, including CD86, iNOS, IL-1β, and IL-6, and promoted microglial polarization toward the M2 phenotype characterized by the upregulation of CD206, Arg-1, Ym-1 and IL-10, in LPS-induced microglia. However, IL-4-silenced aOECs or inclusion of IL-4 NTAb significantly abated the promoting effects of aOECs on M1/M2 polarization, suggesting that IL-4 is likely to be crucially involved in modulating the M2 polarization. To further substantiate this claim, IL-4 was added to LPS-induced microglia to evaluate the shift toward the anti-inflammatory M2 phenotype. Strikingly, IL-4 reduced the proinflammatory cytokine levels and enhanced the polarization of the M2 phenotype. This finding further validated that IL-4 released from aOECs, is a critical mediator of M1 to M2 phenotype conversion. Notably, the promotion of M1/M2 phenotype transformation by IL-4 is weaker than that by aOECs. Therefore, we speculate that there are likely other components in aOECs that synergistically act with IL-4 to drive microglia toward the M2 polarization. Intriguingly, our latest report also demonstrated that aOECs can potentiate the switch of LPS-induced M1 to M2 microglia by upregulating TREM2 in microglia, and the microglial polarization to M2 is partially due to APOE from OECs, which is likely involved in other molecule activity [57]. Thus the finding is strongly supportive of our present view, IL-4 as a central modulator for M1 to M2 microglial phenotype switch. In addition, similar changes were identified by immunofluorescence and immunoblot of several selective specific M1 and M2 markers. These results thus provide a powerful support for IL-4, as a critical cytokine in aOECs that induces M1 to M2 microglial conversion.

Given that endogenous signals from the injury milieu after SCI promote M1 phenotype microglial polarization, lasting pro-M2 anti-inflammatory cytokine stimulation for a persistent M2 phenotypical conversion from an M1 activated state would be a crucial pre-requisite for successful nerve regeneration [24, 58, 59]. Our latest studies revealed that aOECs or their derivatives can effectively promote neural regeneration and functional recovery by modulating microglial polarization to the anti-inflammatory M2 phenotype [24, 28]. This result further prompts us to exactly clarify the key modulators released from aOECs that prevent M1 activation while promoting an M1 to M2 phenotype transformation within the injury environment. Likewise, a similar increase in Arg-1 levels and decrease in iNOS levels with the transplantation of aOECs or delivery of IL-4 after SCI was also observed by immunofluorescence and western blot, and the in vivo M1 to M2 conversion after SCI was accompanied by a significant reduction in the number of M1 type microglia and increase in the number of M2 type microglia, respectively. In SCI model rats injected with IL-4 NTAb and aOECs or IL-4-silenced aOECs, the promotion of M1 to M2 phenotype conversion was greatly abrogated, suggesting that IL-4 is predominantly required for the M1 to M2 microglial phenotype switch albeit a weaker efficacy of IL-4 than aOECs. The results also implied that other components from aOECs and endogenous ligands also provide a powerful synergism with IL-4 to induce M1 to M2 microglial conversion. Therefore, this also accounts for the apparent difference between IL-4 and aOECs in switch of M1/M2 microglial polarization. Regarding IL-4 immunomodulatory effects serving to improve the CNS outcomes after injuries, accumulating evidence indicates that IL-4 serves as an early endogenous neuroprotective agent by anti-inflammatory action and skewing M2 polarization of microglia [60–62]. In view of microglia M2 polarization by IL-4, this is consistent with our finding. Although several previous studies reported that certain anti-inflammatory cytokines, such as IL-10 or TGF-β, also drive the shift of the M1 to M2 phenotype [11, 42, 55, 56, 58–60, 63], the substitution of IL-4 with the above cytokines failed to recapitulate this M1 to M2 microglia transformation (data not shown). In addition, we also demonstrated a similar M1 to M2 transition in both the LPS-induced in vitro and SCI-induced in vivo models of inflammatory insult. The data further substantiated that apart from anti-inflammatory function, IL-4 released from aOECs induces the beneficial traits of M2 microglia/macrophages.

To gain insight into the functional state of M2 polarization from M1 by aOECs, spinal cord neurons were cocultured with LPS-induced microglia in the presence or absence of aOECs, IL-4-silenced aOECs and/or IL-4 NTAb, and IL-4 alone, respectively, and the neuron survival and neurite growth were concomitantly assessed. Excitingly, aOECs significantly curtailed the LPS-induced decrease in neuron viability. Likewise, IL-4 alone also resulted in a slight attenuation of the decreased cell viability, while inclusion of IL-4 NTAb or IL-4-silenced aOECs apparently decreased neuron viability, indicating that these microglia undergoing distinct treatments have acquired genuine M1 or M2 functional profiles. In accordance with this finding, morphological observation and Tju-1 immunostaining in combination with cell apoptotic assessment also indicated that aOECs can effectively attenuate LPS-induced microglial insult to neurons, as demonstrated by enhanced survival of neurons and neurite outgrowth, a dramatic downregulation in expression of C-Caspase-3, and remarkable reduction in the percentage of both necrotic and apoptotic neurons, while inclusion of IL-4 NTAb or IL-4-silenced aOECs effectively counteracted aOEC-promoting activity. In parallel, the inclusion of IL-4 exerts a similar role in protecting neurons from the LPS-induced insults, but is inferior to aOECs. The most likely explanation for these findings is that aOECs contain a variety of bioactive components in addition to IL-4, and that these bioactive components, especially some neural growth factors and neural cell adhesion molecules (NCAMs), also play pivotal roles in promoting cell survival and neurite extension. It is well-known that the pro-inflammatory response following various neural injuries is the major contributor to create a hostile microenvironment and results in expansion of the lesion and further loss of neurologic function [64, 65]. Therefore, the reduction of the inflammatory response facilitates neuronal survival and regeneration by suppressing the activation of resident microglia and macrophages [16]. Based on this point, we speculated that IL-4 secreted by aOECs is a key regulator of microglial M1/M2 polarization to reverse neuroinflammation**.** In addition, our data also revealed that deletion of the IL-4 signal in aOECs leads to a decrease in the number of both necrotic and apoptotic neurons, but the proportion of early apoptotic cells is higher, and the proportion of necrotic cells is less than that of the inclusion of IL-4. This result may imply that the suppressive effect of exogenous IL-4 on proinflammatory insult, to a certain extent, appears to not halt apoptotic cell death resulting from the transition of apoptosis to necrosis, and that other bioactive factors are required for neuron survival and neurite outgrowth. In line with our result, Yang and Taylor et al., also indicates that the anti-inflammatory cytokines IL-4 and TGF β1 exert anti-inflammatory effects, promote M2-like microglial responses, and improve functional recovery after Brain injury [42, 62]. In view of the above findings, we cannot completely exclude the possibility that the system of neurons cocultured with LPS-treated microglia in the presence of aOECs to induce M1 to M2 conversion contains some undetected bioactive factors that lead to release of some other cytokines from M2 polarizing microglia or M2 phenotype to synergistically skew the balance of the microenvironment toward neuroprotection. Nevertheless, several lines of evidence in our study collectively substantiate that the M2 conversion of M1 microglia is modulated by IL-4 released from aOECs.

Based on our present findings, multiple mechanisms appear to be involved in this special combination-mediated M2 phenotypic conversion from an M1 activated state. The molecular mechanism underlying the occurrence of cell events is involved in crosstalk between M2-induced signals and cytokine-modulated signals, mainly activating the JAK1/STAT1/3/6 pathway and NF-κB/SOCS1/SOCS3 cascades, respectively. To gain insight into the signal transduction network responsible for the phenotypic switch of M1 to M2, we have analyzed the functional cross-talk between them. In aOEC-induced M2 phenotypic conversion, we found that IL-4 signaling from aOECs increases the phosphorylation of JAK1/STAT6, and downregulates STAT1/3 by binding of IL-4 to its receptor IL-4Rα. Accumulating evidence indicates that the phosphorylated STAT6 forms a homodimer and translocates into the nucleus, where it binds to specific DNA sequences, thereby regulating the transcription of its target genes responsible for the M2 phenotype and related cytokines [50, 52, 66–73]. Conversely, TLR4 activates the NF-κB signaling pathway, and a predominance of NF-κB and STAT1 activation promotes M1 macrophage polarization, resulting in cytotoxic and damaging proinflammatory functions [50, 73–75]. Furthermore, we found that IL-4 signaling from aOECs can significantly inhibit LPS-induced the increase in phosphorylated NF-κB, suggesting that IL-4 signaling from aOECs is likely to impair NF-κB activation through the JAK1/STAT6 signaling pathway. Strikingly, in our study, we found that LPS also increased phosphorylated STAT1/STAT3 levels, and that deletion of IL-4 signaling increased phosphorylated STAT1/STAT3 levels, implying that IL-4 is a key modulator of microglial polarization to the M2 phenotype. It has been demonstrated that TLR signaling skews microglia/macrophages toward the M1 phenotype via STAT1, and the negative regulatory function of SOCS3 in microglia by inhibiting STAT1 and STAT3 activation [72–75]. The report is in line with our present finding, indicting the heterogeneity of STAT3 biofunctions in the cellular events.

SOCS proteins function as feedback inhibitors for cytokines that use the JAK/STAT pathway, and can be directly induced by TLR signaling [49, 50]. Among them, SOC1 and SOCS3 function as a modulators of microglia/macrophage polarization, limiting excessive cytokine release resulting from persistent activation of STAT3 [43, 50, 76]. Furthermore, the influence of SOCS3 on M1 and M2 microglia mainly includes activators, inducers, and transducers, etc. [43, 50]. In this study, we have evaluated the polarization of microglia from LPS-induced microglia with or without aOECs. We found that microglia treated with LPS significantly upregulated the expression of SOCS1 and SOCS3, while aOECs downregulated their expression. Interestingly, IL-4 coincubation with LPS inhibited SOCS1 expression. For SOCS3, IL-4 appears to considerably influence SOCS3 expression. In view of SOCS3 as a modulator of macrophage activation and M1 polarization via the STAT signaling pathway, we speculate that SOCS1/SOCS3 signaling is also involved in the molecular networks orchestrating the switch of microglial polarization.

**Fig. 9:**
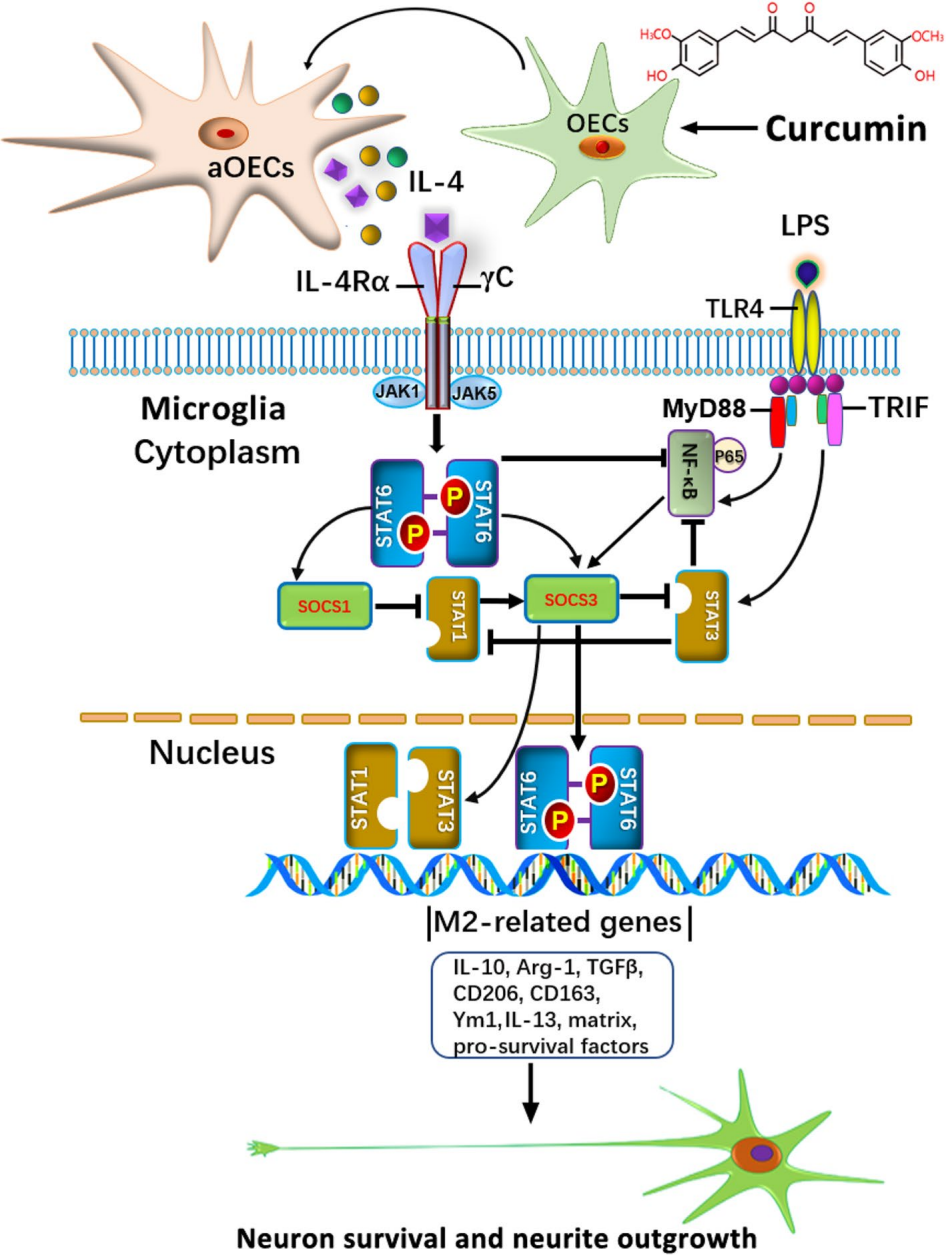
Schematic diagram showing the underlying molecular mechanism by which aOECs suppress LPS-induced neuroinflammation by regulating the switch of M1 to M2 microglial phenotypes mainly via IL-4 mediation

To further authenticate the involvement of the aforementioned pathways in the switch of M1 to M2 during the progression of inflammatory responses, several selective antagonists AZD1402, AS1517499 and LY300194, were used to detect microglial M1/M2 polarization, respectively, following LPS induction in the presence of aOECs. As expected, these antagonists, to a considerable extent, upregulated the expression of CD86 and iNOS, which are protective of the M1 phenotype, by suppressing the acquisition of protective M2 traits (CD206 and Arg-1). Our findings demonstrate that the promotion of microglial M2 polarization through the IL-4Rα/JAK1/STAT6 pathway participates in IL-4-mediated anti-inflammatory effects after aOEC induction. The result is also consistent with Qin’s report implicating M2 macrophage polarization enhanced by IL-4 through JAK1 and STATS signals [71]. This evidence further supports a potential role of IL-4 from activated OEC in microglial M1/M2 polarization. To further confirm whether inhibiting the critical signaling pathways responsible for microglial M2 polarization truly causes abortion in the switch from M1 to M2 phenotypes, we cocultured neurons with LPS-stimulated microglia treated with aOECs in the presence of the aforementioned antagonists. Our results revealed that disruption of IL-4Rα/Jak1/STAT6 biofunctions effectively abrogated M2 phenotypic conversion from an M1 activated state and the further promoting effects on the neurons, exhibiting a dramatically reduced cell survival rate and neurite extension, as well as severely disintegrated neurites, implying that IL-4Rα/JAK1/STAT6 signaling cascades are involved in IL-4-mediated cellular events. Although the exact mechanisms responsible for microglial M1/M2 phenotypic switching modulated by aOECs remain to be elucidated, the present results indicate that there is a molecular cross-talk between the JAK1/STAT1/3/6 and NF-κB/SOCS1/SOCS3 cascades, which orchestrates the switch of microglia M1/M2 by IL-4 signals released from aOECs. More importantly, considering the possibility of synergistic involvement of other signaling molecules produced by aOECs, the underlying crosstalk needs to be further analyzed for a full understanding of the M1–M2 paradigm of microglial polarization. Coincidentally, our latest study substantiated the present claim. In short, the underlying mechanisms of the switch of microglia M1/M2 by IL-4 signals released from aOECs were illustrated in Fig. 9. In addition, the aOECs exert a stronger regeneration-promoting effect on the injured spinal cord than normal OECs and other treatment groups. This is in agreement with our previous report regarding the enhancement of aOECs on spinal cord regeneration [24, 28], thus implying the contribution of aOECs to the improvement of micro-environment around the injured region by phenotypic switching from M1 to M2 microglia aside with the elevated neurotrophins.

## Conclusions

To the best of our knowledge, this study is the first report that IL-4 released from aOECs functions as a master modulator of microglial M1/M2 polarization, which, in turn, effectively promotes neuron survival and neurite outgrowth. This also implies a strengthened anti-inflammatory capacity of OECs once activated. Furthermore, we also demonstrate that the crosstalk between the JAK1/STAT1/3/6 signaling pathways and NF-κB/SOCS1/SOCS3 cascades orchestrates IL-4-mediated M2 polarization of microglia. The beneficial role of aOECs in restricting inflammatory damage provides novel insights into the treatment of SCI and a variety of neural degenerative diseases.

### Supplementary Information

**Additional file 1: S1 Figure.** Detection of knockdown of IL-4 expression in aOECs. (a) Representative western blot showing that IL-4 protein level was reduced in aOECs transfected with IL-4 siRNA. (b) Release of IL-4 from aOECs after knockdown of IL-4 gene by ELISA. (c) The effect of transfection with IL-4 siRNA on the cell viability by MTT. **S2 Figure.** The quantification of Arg-1 positive microglia/macrophages at the injury and the injection area. **Table 1.** Primer sequences of siRNAs. **Table 2.** List of primers used for real-time reverse transcription polymerase chain reaction.

**Additional file 2.**

## Data Availability

The data that support the findings of this study are available from the corresponding author upon reasonable request.

## Abbreviations

IL-4: Interleukin-4
Arg-1: Arginase-1
CD206: Macrophage mannose receptor
CCM: Curcumin
NTAb: Neutralizing antibody
TG2: Transglutaminase-2
PSR: Phosphatidylserine receptor
CD86: Recombinant cluster of differentiation 86
iNOS: Inducible nitric oxide synthase
CXCL1: C-X-C motif Chemokine ligand-1
TLR4: Toll-like receptor 4
Ym-1: Chitinase-like 3
VEGF: Vascular endothelial growth factor
PDGF: Platelet-derived growth Factor
IFN-γ: Interferon gamma
TNF-α: Tumor necrosis factor-α
GDNF: Glial cell line-derived neurotrophic factor
JAK1: Janus kinase-1
STAT: Signal transducer and activator of transcription
SOCS: Suppressor of cytokine signaling
