# Supplementary material for: Interleukin-4 from curcumin-activated OECs emerges as a central modulator for increasing M2 polarization of microglia/macrophage in OEC anti-inflammatory activity for functional repair of spinal cord injury
Source: Cell Commun Signal. 2024 Mar 6;22:162. doi: 10.1186/s12964-024-01539-4 (PMC10916222; doi:10.1186/s12964-024-01539-4)
Supplement: Supplementary file 1 — Additional file 1: S1 Figure. Detection of knockdown of IL-4 expression in aOECs. (a) Representative western blot showing that IL-4 protein level was reduced in aOECs transfected with IL-4 siRNA. (b) Release of IL-4 from aOECs after knockdown of IL-4 gene by ELISA. (c) The effect of transfection with IL-4 siRNA on the cell viability by MTT. S2 Figure. The quantification of Arg-1 positive microglia/macrophages at the injury and the injection area. Table 1. Primer sequences of siRNAs. Table 2. List of primers used for real-time reverse transcription polymerase chain reaction. [file 12964_2024_1539_MOESM1_ESM.docx]

**Supplement data:**

**S1 Figure. Detection of knockdown of IL-4 expression in aOECs.** (a) Representative western blot showing that IL-4 protein level was reduced in aOECs transfected with IL-4 siRNA. (b) Release of IL-4 from aOECs after knockdown of IL-4 gene by ELISA. (c) The effect of transfection with IL-4 siRNA on the cell viability by MTT

**S2 Figure. The quantification of Arg-1 positive microglia/macrophages at the injury and the injection area.**

**Table 1. Primer sequences of siRNAs**

**Table 2. List of primers used for real-time reverse transcription polymerase chain reaction.**

**S1 Figure**

**
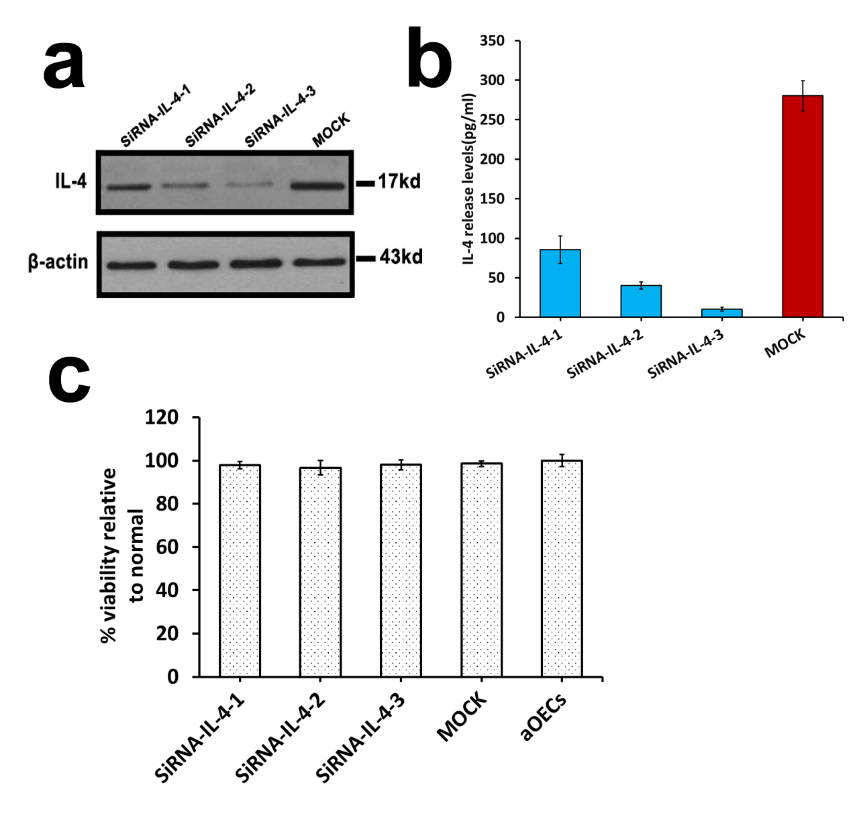
**

**S2 Figure**

**
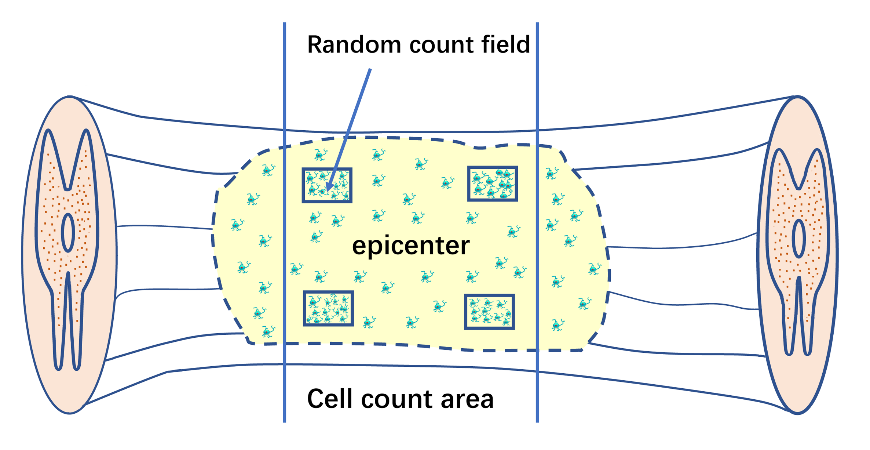
**

**Table**

**Table 1 List of primers used for real-time reverse transcription polymerase chain reaction**

**Table 1 Primer Sequences used for RT-qPCR analysis**

| **Target gene** | **Forward primer sequence (5’-3’)** | **Reverse primer sequence (5’-3’)** |
| --- | --- | --- |
| **CD86** | TAGGGATAACCAGGCTCAC | CGTGGGTGTCTTTTGCTGTA |
| **iNOS** | GCAGAATGTGACCATCATGG | ACAACCTTGGTGTTGAAGGC |
| **IL-1β** | TGATGTTCCCATTAGACAGC | GAGGTGCTGATGTACCAGTT |
| **IL-6** | TCTTGGGACTGATGCTGGTG | CAGAATTGCCATTGCACAACTC |
| **CD206** | AGTTGGGTTCTCCTGTAGCCCAA | ACTACTACCTGAGCCCACACCTGCT |
| **Arg-1** | TCACCTGAGCTTTGATGTCG | TTCCCAAGAGTTGGGTTCAC |
| **IL-10** | AATTCCCTGGGTGAGAAGCTG | TCATGGCCTTGTAGACACCTTG |
| **Ym1** | ACCCCTGCCTGTGTACTCACCT | CACTGAACGGGGCAGGTCCAAA |

**Table 2 Primer sequences of siRNAs**

| **Target gene** | **Sequence of sense strand (5’-3’)** |
| --- | --- |
| **shRNA1-IL4** | AAGCTGCACCATGAATGAGTC |
| **shRNA2-IL4** | AACACCACAGAGAGTGAGCTC |
| **shRNA3-IL4** | AATGTACCAGGAGCCATATCC |
