# Supplementary figures and images for: Interleukin-4 from curcumin-activated OECs emerges as a central modulator for increasing M2 polarization of microglia/macrophage in OEC anti-inflammatory activity for functional repair of spinal cord injury
Source: Cell Commun Signal. 2024 Mar 6;22:162. doi: 10.1186/s12964-024-01539-4 (PMC10916222; doi:10.1186/s12964-024-01539-4)

**IL-4**

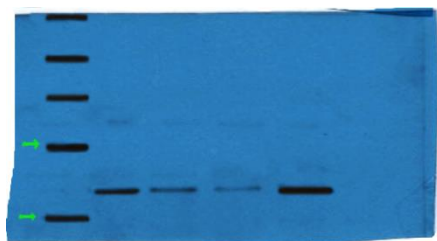

**$\beta$ -actin**

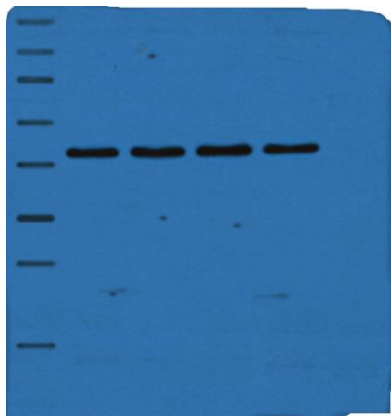

Supplement: Supplementary file 2 — Additional file 2. [file 12964_2024_1539_MOESM2_ESM.zip › 1-Supplement data.pdf]
